# MESH1 knockdown triggers proliferation arrest through TAZ repression

**DOI:** 10.1038/s41419-022-04663-6

**Published:** 2022-03-10

**Authors:** Tianai Sun, Chien-Kuang Cornelia Ding, Yuning Zhang, Yang Zhang, Chao-Chieh Lin, Jianli Wu, Yasaman Setayeshpour, Si’Ana Coggins, Caitlin Shepard, Everardo Macias, Baek Kim, Pei Zhou, Raluca Gordân, Jen-Tsan Chi

**Affiliations:** 1 Department of Molecular Genetics and Microbiology, Duke University, Durham, NC 27710 USA; 2 Center for Genomic and Computational Biology, Duke University, Durham, NC 27708 USA; 3 Program in Computational Biology and Bioinformatics, Duke University, Durham, NC 27708 USA; 4 Center for Drug Discovery, Department of Pediatrics, School of Medicine, Emory University, Atlanta, GA 30322 USA; 5 Department of Pathology, Duke University, Durham, NC 27708 USA; 6 Department of Biochemistry, Duke University School of Medicine, Durham, NC 27710 USA; 7 Department of Biostatistics and Bioinformatics, Duke University Medical Center, Durham, NC 27710 USA; 8 Department of Computer Science, Duke University, Durham, NC 27708 USA

**Keywords:** Cancer genetics, Cell growth, Cell signalling

## Abstract

All organisms are constantly exposed to various stresses, necessitating adaptive strategies for survival. In bacteria, the main stress-coping mechanism is the stringent response triggered by the accumulation of “alarmone” (p)ppGpp to arrest proliferation and reprogram transcriptome. While mammalian genomes encode *MESH1*—the homolog of the (p)ppGpp hydrolase SpoT, current knowledge about its function remains limited. We found MESH1 expression tended to be higher in tumors and associated with poor patient outcomes. Consistently, *MESH1* knockdown robustly inhibited proliferation, depleted dNTPs, reduced tumor sphere formation, and retarded xenograft growth. These antitumor phenotypes associated with *MESH1* knockdown were accompanied by a significantly altered transcriptome, including the repressed expression of *TAZ*, a HIPPO coactivator, and proliferative gene. Importantly, TAZ restoration mitigated many anti-growth phenotypes of *MESH1* knockdown, including proliferation arrest, reduced sphere formation, tumor growth inhibition, dNTP depletion, and transcriptional changes. Furthermore, TAZ repression was associated with the histone hypo-acetylation at TAZ regulatory loci due to the induction of epigenetic repressors HDAC5 and AHRR. Together, *MESH1* knockdown in human cells altered the genome-wide transcriptional patterns and arrested proliferation that mimicked the bacterial stringent response through the epigenetic repression of *TAZ* expression.

## Introduction

All organisms are constantly exposed to a wide variety of environmental stresses. For example, most solid tumor cells experience hypoxia [1, 2], lactic acidosis [3, 4], and nutrient deprivations [5–9], necessitating stress responses to ensure survival and homeostasis. In bacteria, the main stress mechanism is “stringent response” mediated by elevated alarmone (p)ppGpp via synthesis by its synthetase (RelA), or inhibited degradation by its hydrolase (SpoT). The accumulated (p)ppGpp triggers transcriptional reprogramming and proliferation arrest [10] through suppression of *de novo* dNTP synthesis [11, 12]. While a similar stress response has not been reported in metazoan, metazoan genomes encode *MESH1* (Metazoan SpoT Homolog 1, also named HDDC3). The difficulties are embedded in the fact that neither (p)ppGpp nor RelA exists in metazoan cells. A previous study [13] described the conserved structures of Drosophila and human MESH1 and their in vitro enzymatic activities to degrade (p)ppGpp. Genetic depletion of *Drosophila* MESH1 enhanced stress survival and triggered transcriptional responses bearing significant similarities to bacterial stringent response [13]. Recently, we have shown that MESH1 is a cytosolic NADPH phosphatase and *MESH1* knockdown protected cancer cells against ferroptosis [14] and triggered integrative stresses response. However, much remains unknown about the phenotypic responses to *MESH1* knockdown.

Here, we report that *MESH1* knockdown robustly arrests proliferation, depletes dNTP, inhibits tumor spheres, and xenograft growth. Mechanistically, these anti-growth effects are mediated by the transcriptional repression of HIPPO effector *TAZ* (encoded by *WWTR1*, WW domain containing transcription regulator 1), known to regulate proliferation and tumorigenesis. In addition, the transcriptional repression of *TAZ* is associated with a repressive chromatin modulation by the induction of HDAC5 (histone deacetylase 5) and AHRR (aryl-hydrocarbon receptor repressor). The regulation of *HDAC5, AHRR, TAZ*, and cell proliferation depends on the enzymatic activities of MESH1. Together, our results indicate that *MESH1* knockdown in human cells triggers proliferation arrest through epigenetically repressing *TAZ* transcription, and may hold antitumor therapeutic potential.

## Results

### *MESH1* knockdown reduced cell number, proliferation, and tumor growth

**Fig. 1:**
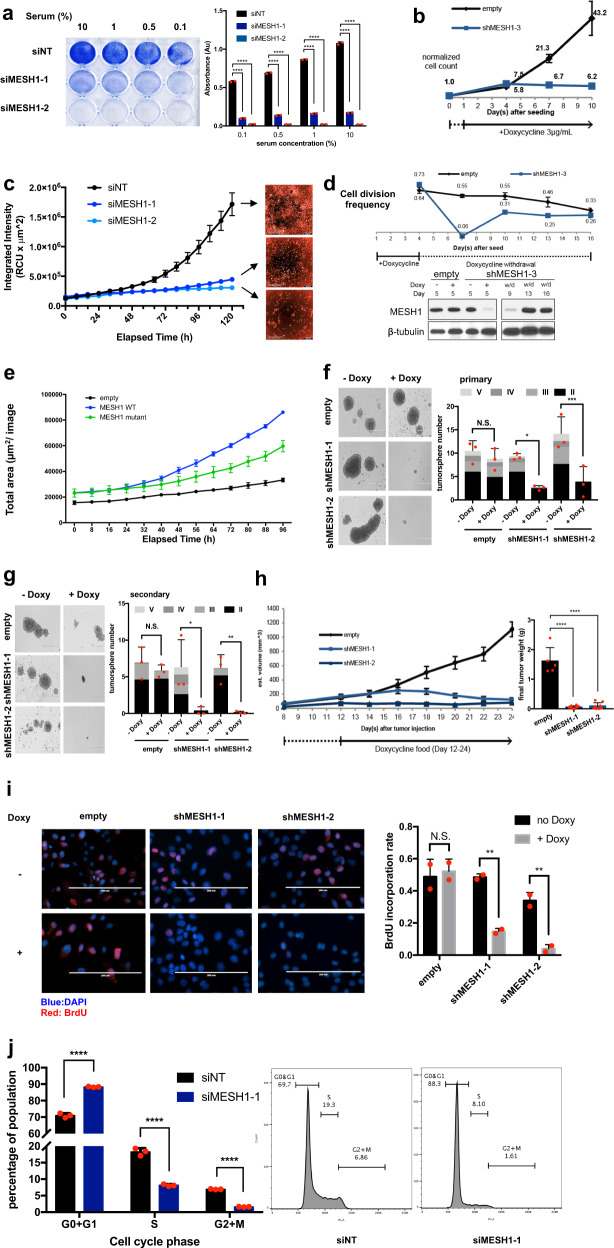
*MESH1* knockdown led to cell proliferation arrests and delayed xenograft growth. **a** Representative image of the crystal violet staining (left) and quantification for replicates (right) of H1975 cells under different serum concentrations showed consistent cell number reduction upon *MESH1* knockdown. Higher absorbance represents higher cell numbers. (mean + s.d.). **b** Cell number measurement by hemocytometer on Days 4, 7, and 10 revealed reduced cell number upon *MESH1* knockdown by doxycycline-inducible shRNA of *MESH1* in RCC4 cells. (mean ± s.d.). **c** Image-based quantification of cell count and representative fluorescence-labeled images (right) after 5 days revealed that *MESH1* knockdown significantly reduced cell numbers. H1975 were stably transduced with histone H2B-mcherry before being transfected with the indicated siRNAs. Images and fluorescence intensity were obtained and quantified by the Incucyte S3 every 8 h for 5 days after siRNA treatment. Fluorescence intensity (quantified by the Incucyte S3) was proportional to the proliferating cell number. (mean ± s.d.). **d** Cell number measurement by hemocytometer on Days 4, 7, 10, 13, and 16 revealed that cell number reduction upon doxycycline-induced MESH1 knockdown in RCC4 cells was reversible when doxycycline was withdrawn and MESH1 protein was reexpressed confirmed by the Western blot. Estimated cell division frequency per 24 h were calculated based on cell count (log2(fold change)/days). (mean ± s.d.). **e** Image-based quantification of fluorescence intensity by Incucyte S3 in RCC4 revealed that wild-type *MESH1* expression largely stimulated cell growth. The enzymatic dead mutant *MESH1* had a reduced capacity to enhance cell growth (mean ± s.d.). **f** Representative image (left) and quantification (right) of the primary tumor sphere formation assay revealed that *MESH1* knockdown reduced both the number and the size of H1975 tumor spheres. Colors of bars from lighter to darker successively represent tumor spheres with diameters of >400 μm (V), 300–400 μm (IV), 200–300 μm (III), and 100–200 μm (II). (mean + s.d.). **g** Representative image (left) and quantification (right) of the secondary tumor sphere formation assay confirmed the same reduction in number and size of H1975 tumor spheres by *MESH1* knockdown. The colors of bars represent the same category as (**e**). (mean + s.d.). **h** Tumor size and weight measurement showed xenograft growth inhibition upon doxycycline-induced *MESH1* knockdown in the xenografted tumor model. *p* value was calculated by the one-way ANOVA followed by Tukey’s posttest. (mean + s.d.). **i** Representative image and quantification for the BrdU incorporation assay before and after the doxycycline-induced *MESH1* knockdown in H1975 revealed a significant reduction of the BrdU incorporation rate upon *MESH1* knockdown. Scale bars: 200 μm. (mean + s.d.). **j** Cell cycle distribution of H1975 upon *MESH1* knockdown by PI stain. The percentage of cells in each individual stage was calculated by the FlowJo software and labeled in the histograms correspondingly. Bar graphs represent the average percentage of cells in each individual stage in each group. siMESH1 reduced the portions of both S and G2 + M phases and arrested cells in the G0 + G1 phase. (mean + s.d.). For **a**; **f**; **g**; **i**; **j**, *p* values were calculated by the two-way ANOVA followed by Tukey’s posttest. *P~(0.01, 0.05); **P~(0.001, 0.01); ***P~(0.0001, 0.001); ****P < 0.0001; NS no significance.

The polymorphism in the 3′UTR of *MESH1* was found associated with a risk of breast cancers, implying its relevance to tumor biology [15]. Interestingly, TCGA datasets revealed that *MESH1* was generally expressed at higher levels in tumor than non-tumor tissues (Supplementary Fig. 1a). Therefore, we investigated the phenotypic response of cancer cells to *MESH1* knockdown by multiple siRNAs (Supplementary Fig. 1b, c). *MESH1* knockdown significantly reduced the cell number of non-small cell lung cancer (H1975) (Fig. 1a) under different serum levels tested by crystal violet staining. We expanded this finding in a broader panel of cancer cell lines, including renal cell carcinoma (RCC4, 786-O), breast cancer (BT20, BT474, MCF-7), chondrosarcoma (SW-1353), and fibrosarcoma (HT-1080), and noticed a consistent cell number reduction by *MESH1* knockdown (Supplementary Fig. 1d). The antitumor effects of *MESH1* knockdown in H1975, RCC4, and 293 T were further confirmed using CellTiter-Glo assay (Supplementary Fig. 1e). In essence, the effects were specific since they were abolished by the reexpression of the wild-type MESH1 (Supplementary Fig. 1f). Interestingly, the enzymatic dead mutant MESH1 did not restore the cell number (Supplementary Fig. 1f). And the knockdown of NAD(H) kinase (NADK), mediating the NADPH generation opposite to MESH1’s activity, largely mitigated the reduced cell growth (Supplemental Fig. 1g). These findings indicated the importance of MESH1 NADPH phosphatase activity in regulating cell growth. We further validated that *MESH1* knockdown by doxycycline (Doxy)-inducible shRNAs (Supplementary Fig. 1h, i) also triggered growth inhibition tested by cell counting (Fig. 1b) and the Incucyte^®^ S3 over time (Fig. 1c and Video 1a–c). Importantly, upon the Doxy withdrawal and restoration of MESH1 expression, cell growth resumed (Fig. 1d). Reciprocally, wild-type MESH1 expression enhanced proliferation, while the effect of the enzymatic dead mutant MESH1 was much reduced (Fig. 1e).

Next, we tested the anti-growth effects of *MESH1* removal in 3D tumor spheres and xenografts. The doxycycline-induced *MESH1* knockdown significantly reduced the number and size of tumor spheres (Fig. 1f). We then dissipated primary spheres and seeded the same number of cells for the secondary assays, which further confirmed the continuous suppression of the tumor sphere formation by *MESH1* knockdown (Fig. 1g). Similar results were obtained in vivo using subcutaneous H1975 xenografts. The induction of two *MESH1* shRNAs significantly reduced the volume and weight of tumors (Fig. 1h). Collectively, inducible *MESH1* knockdown inhibited tumor growth both in vitro and in vivo.

Mechanistically, we investigated whether *MESH1* knockdown (1) inhibited cell proliferation (BrdU incorporation assay), or (2) enhanced cell death (CellTox Green assay) to reduce cell number. *MESH1* knockdown significantly reduced the BrdU incorporation (proliferation) (Fig. 1i) but did not increase cell death (Supplementary Fig. 1j). Additionally, the propidium iodide (PI) stain suggested that *MESH1* knockdown inhibited the G1-S phase transition (Fig. 1j) without increasing the sub-G1 population, supporting the lack of significant cell death. Collectively, *MESH1* knockdown inhibited cell proliferation to achieve anti-growth effects.

### The prognostic significance of MESH1 in tumor expression datasets

Based on the antitumor phenotypes associated with *MESH1* knockdown, we analyzed the prognostic significance of *MESH1* (*HDDC3*) expression in the tumor datasets. Results suggested that lower *MESH1* mRNA expression was associated with significantly better survivals in patients with renal cell carcinoma (RCC) (Supplementary Fig. 2a), clear cell type RCC (TCGA) (Supplementary Fig. 2b), lung cancers (Supplementary Fig. 2c, d) [16], neuroblastoma (GSE62564) (Supplementary Fig. 2e), colon cancer (GSE38832) [17] (Supplementary Fig. 2f), meningioma (GSE16581) [18] (Supplementary Fig. 2g), and follicular lymphoma (GSE16131) [19] (Supplementary Fig. 2h). These in vivo associations of low *MESH1* levels and better clinical outcomes suggested a functional role of MESH1 in tumor biology.

### Transcriptional responses and dNTP depletion upon *MESH1* knockdown

**Fig. 2:**
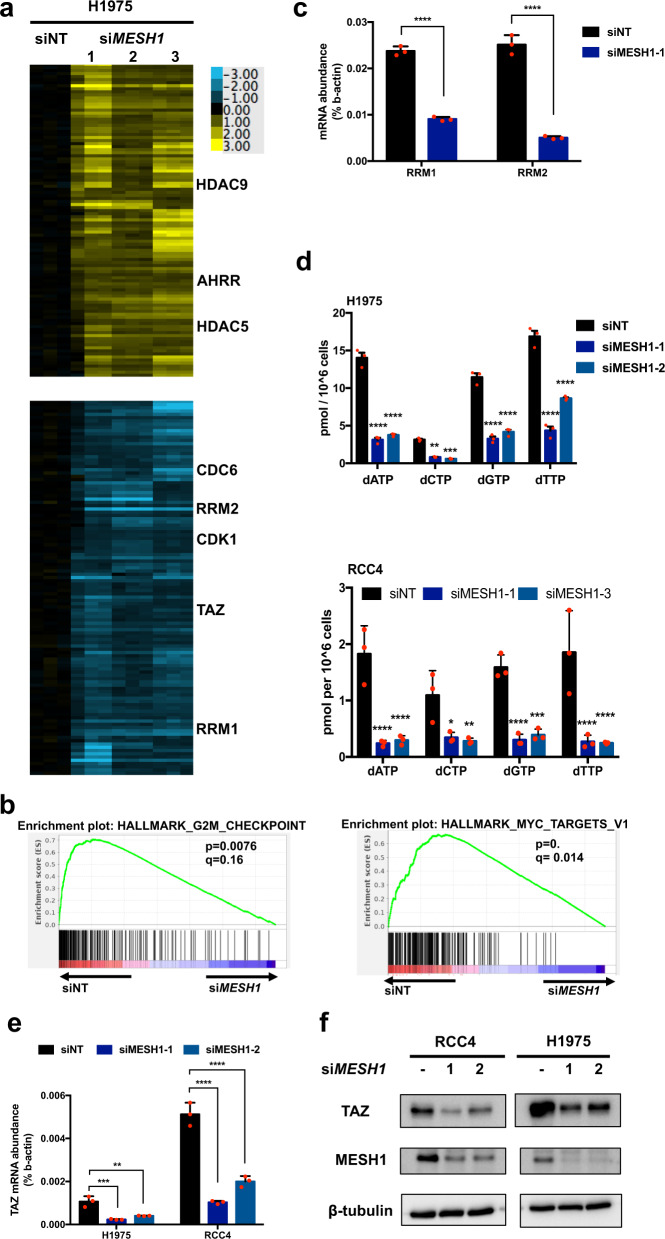
The transcriptional response to *MESH1* knockdown. **a** Heatmap of the selected genes whose expressions were significantly affected by all three MESH1-targeting siRNAs in H1975 cells. Cells were treated with the nontargeting siRNA or three distinct *MESH1* targeting siRNAs and triplicates for each treatment were collected. Filtering criteria resulted in 336 probesets: at least seven observations with absolute value ≥0.65. **b** GSEA analysis of the inhibition of cell cycle-related and Myc-targeted genesets in H1975 cells upon *MESH1* knockdown. Gene signatures were downloaded from the GSEA Molecular Signatures Database. Hallmark_G2M _Checkpoint: *p* = 0.0076, *q* = 0.16; Hallmark_Myc_Targets: *p* = 0, *q* = 0.014. **c** qRT-PCR validation of the reduced *RRM1* and *RRM2* mRNA in the *MESH1*-knockdown H1975 cells. (mean + s.d.)**. d** dNTP measurement by MS-based analysis shows that *MESH1* knockdown reduced the levels of all four measured dNTPs in H1975 and RCC4. (mean + s.d.)**. e** qRT-PCR validation of the repressed *TAZ* mRNA expression upon *MESH1* knockdown in H1975 and RCC4 cells. (mean + s.d.)**. f** Representative image of the western blot, which validated that *MESH1* knockdown repressed TAZ protein levels in H1975 and RCC4 cells. For **c**, *p* values were calculated by the two-tail student’s *t*-test. For **d**, *p* values were calculated by the two-way ANOVA followed by Tukey’s posttest. For **e**, *p* values were calculated by the one-way ANOVA followed by Tukey’s posttest. **P~(0.001, 0.01); ***P~(0.0001, 0.001); ****P < 0.0001.

One prominent feature of bacterial stringent response is transcriptional reprogramming [10, 20]. *MESH1* removal in *Drosophila* downregulated the DNA and protein synthesis-related gene expression [13]. Therefore, we analyzed the global transcriptional responses to *MESH1* knockdown (Fig. 2a) in H1975 cells. 994 genes were selected based on the filtering criteria of at least seven observations with absolute log_2_ values >0.47, and arranged by clustering (Fig. 2a) and the differentially expressed genes were shown in Table 1. Gene Set Enrichment Analysis (GSEA) indicated that *MESH1* knockdown inhibited multiple cell cycle progression-related pathways (Fig. 2b), including repressing the expression of *CDC6* (cell division cycle 6), and *CDK1* (cyclin-dependent kinase 1) (Fig. 2a), consistent with the proliferation arrest phenotypes (Fig. 1). *MESH1* knockdown also induced the expression of *HDAC5 and HDAC9* (histone deacetylases), implicating epigenetic modulations. Additionally, *MESH1* knockdown repressed the expression of *RRM1* (ribonucleotide reductase M1) and *RRM2* (Fig. 2c), two subunits of ribonucleotide reductase (RNR) responsible for de novo synthesis of dNTPs [21, 22]. As ppGpp depletes dNTPs in bacteria [12], we measured the dNTP levels in H1975 and RCC4 (Fig. 2d) and found that *MESH1* knockdown largely reduced the levels of all four dNTPs. Together, these results suggested that *MESH1* knockdown depleted dNTPs and repressed the expression of genes regulating dNTP synthesis and cell cycle progression.

**Table 1:** The list of differentially expressed genes upon MESH1 knockdown.

Gene Symbol
NREP
NREP
HDAC5
SYT1
CA11
AKR1C3
PCDHA1 /// PCDHA10 /// PCDHA11 /// PCDHA12 /// PCDHA13 /// PCDHA2 /// PCDHA3 /// PCDHA4 /// PCDHA5 /// PCDHA6 /// PCDHA7 /// PCDHA8 /// PCDHA9 /// PCDHAC1 /// PCDHAC2
ZNF467
KLRC3
GSN
ARG2
DDAH1
NMNAT2
CDC14B
DIP2C
DZANK1
RUNDC3A
STK19
HIST1H2BC /// HIST1H2BE /// HIST1H2BF /// HIST1H2BG /// HIST1H2BI
HIST1H2BE
HIST1H2BD
HIST1H2BC /// HIST1H2BE /// HIST1H2BF /// HIST1H2BG /// HIST1H2BI
IFT22
INPP5A
SIK1
CFB
ATF3
RHOD
KLK6
MGLL
UAP1L1
DSP
KLHDC3
KLHDC3
GOLGB1
DDAH2
DDAH2
DDAH2
TPM4
KDM2A
CCDC176
FLRT2 /// LOC100506718
CCPG1 /// DYX1C1-CCPG1
CCPG1 /// DYX1C1-CCPG1
RRAGD
LCAT
ZBTB5
CFDP1
IQCJ-SCHIP1 /// SCHIP1
SAT1
SAT1
SAT1
CASP7
UBE2L6
RABAC1
OAZ3
C11orf80
NABP1
ITGB5
PLAG1
CXADR
COL18A1
PIM1
MCCC1
OPTN
VAMP5
ATP9A
HABP4
ZER1
CDC14B
HBP1
TBC1D9
UBAP2L
AKR1A1
BBS1
CTSB
E2F3
EHD1
EHD1
EHD1
DNAJC1
ARID3A
CCDC93
CARS
RRAGD
TMEM43
DYNC1H1
TTC9
CARHSP1
SIGIRR
SIGIRR
SPCS3
LPIN2
LTBP1
ITGB5
HDAC9
C11orf95
ADCY9
SLC2A3
DNAJB9
CTSB
SLC2A3
CTSB
EDEM1
SLC2A14 /// SLC2A3
KLF9
AGR2
ZNF83
G3BP2
ZNF267
PHACTR2
ACYP2 /// LOC101927144
PAEP
VPS28
CREBL2
DLG5
ANKRA2
KIAA1598
2-Mar
HIST1H1C
CDKN1C
CDKN1C
---
CDKN1C
CDKN1C
AHNAK2
CREBL2
IFT20
FOS
KDR
KCNJ15
DPYSL3
MTF2
RSL1D1
PHTF2
RSL1D1
HIST1H2BC /// HIST1H2BE /// HIST1H2BF /// HIST1H2BG /// HIST1H2BI
ANGPTL4
IL24
OSTM1
MAFF
DUSP3
PHACTR2
RGL2
STX4
SPAG7
TUSC3
CTSB
CDYL
NUPL1
BIK
EFTUD1
RAB17
PLEKHA1
KAT2B
ZNF702P
LY96
CHIC2
MAPK6
TLK2
CBY1
EMC6
CDC37L1
IL6R
CREB3
ARL14
MUT
JUN
HIST1H4H
IFT88
HIST1H2AG /// HIST1H2AH /// HIST1H2AI /// HIST1H2AK /// HIST1H2AL /// HIST1H2AM
BSPRY
HIST1H2BG /// HIST1H2BJ
HIST1H2AE
BICD2
HIST2H2AA3 /// HIST2H2AA4
HIST2H2AA3 /// HIST2H2AA4
LINC00339
S100A13
PSENEN
CCDC53
AHNAK
DDX43
C2orf54
MAD2L1
CCT2
MCM6
PLK1
GTSE1 /// TRMU
EPB41L2
ACOX2
ACLY
ABCE1
EVI2B
SRSF1
LHX6
ACLY
EOGT
PRPS1
KIF14
MIR636 /// SRSF2
RAD54B
RFWD3
MDFIC
NAA50
MIS18BP1
SLC29A1
STRAP
MRTO4
TMPO
RAC2
HNRNPH1
H2AFX
IL1RL1
TUBGCP3
UBE2D2
ARHGAP22
RAB28
KPNA4
PARN
DUSP9
TLE3
FBXO11
NBN
HIP1
RGS4
GJA9-MYCBP /// MYCBP
HNRNPA2B1
DAZAP1
ARTN
ARTN
ARTN
PPP6R3
RBM8A
NHLRC2
WDR77
WWTR1
PRR3
IDH3A
PRPF4
NAA15
ARF6
HIPK2
IL1RN
C6orf62
STIP1
BCLAF1
BCLAF1
NBN
WWTR1
PIGL
DHX15
SERBP1
MIR4745 /// PTBP1
SMC4
GPR107
BUB1
ENO1
PRKAR2B
CD44
LOC101928747 /// RBMX /// SNORD61
DARS2
CEP152
SRSF11
BCLAF1
TRIM14
TRIM14
MBNL1
TMED2
ARF1 /// MIR3620
TUBB2A /// TUBB2B
STC1
STC1
CSNK2A1
LPAR1
RBM12
ZNF586
HNRNPD
SORD
SORD
BASP1
PDHA1
HNRNPD
6-Mar
KIAA1462
PRMT3
NT5DC2
PTGES
C6orf62
PRKX
TIA1
H2AFV
H2AFV
FAM115A /// LOC100294033
FAM115A /// LOC100294033
ELAVL1
ALDH3A2
ALDH1A3
KRAS
ARMC9
ZNF207
GPR125
ADO
CYB5B
DESI1
LIPG
GTPBP8
SDHD
LRRC59
MRPL44
GPRC5B
SCLY
FUBP1
ANKLE2
QRSL1
AMACR /// C1QTNF3-AMACR
SPATS2L
MALL
PSME3
HNRNPUL1
NAP1L1
OPA1
PPP2R1B
TRIM14
LRRK1
ACTR3B
HNRNPUL1
MAP3K7
ACSL3
ACSL3
SEC23IP
ARHGEF26
ALDOC
METAP1
POT1
FASTKD2
PUS7
GATC
IL18
CALML4
CALML4
TIA1
NAP1L1
RRP15
PEG10
CA2
ARHGAP29
ACTB
FCF1
ABLIM1
THEMIS2
U2SURP
PAPOLA
HHEX
METAP2
PTER
DLG1
TAF6L
FAH
EVI2A
NETO2
CDK1
CDC25C
CDC6
SRSF6
GINS1
FADS1 /// MIR1908
FADS1 /// MIR1908
FADS1 /// MIR1908
CBLL1
NRP1
DKK1
VDAC1
FUS
TBCE
CKB
AASDHPPT
HIRA
ATP2A2
STARD7
WDR3
MOCOS
LRRC40
GEMIN2
AIDA
RRM2
RRM1
RRM1

### *MESH1* knockdown repressed *TAZ* mRNA expression that contributed to the antitumor phenotypes

Interestingly, we noted consistent repression of *WWTR1* (WW domain containing transcription regulator, which encodes TAZ (transcriptional coactivator with the PDZ-binding motif) (Fig. 2a). TAZ and its paralog YAP (Yes-associated protein) are well-recognized HIPPO effectors that regulate proliferation and self-renewal of cancer cells [23, 24]. While YAP/TAZ are conventionally co-regulated by protein phosphorylation, and translocation [23, 24], *MESH1* knockdown reduced *TAZ* mRNA level in all tested cancer cells (Fig. 2e, f and Supplementary Fig. 3a). Importantly, wild-type, but not mutant, *MESH1* restoration mitigated *TAZ* repression, showing the specificity (Supplementary Fig. 3b). The rescue by *NADK* knockdown further reiterated the importance of MESH1 enzymatic activity (Supplementary Fig. 3c). While TAZ levels are affected by cell density at the posttranslational level [25–28], we found that *MESH1* knockdown reduced *TAZ* mRNA to comparable degrees in H1975 and RCC4 grown with low, medium, or high cell density (Supplementary Fig. 3d). Similarly, *MESH1* knockdown did not alter the nuclear/cytosolic distribution of TAZ measured by fractionation/Western blots (Supplementary Fig. 3e) and immunofluorescence (Supplementary Fig. 3f). Consistently, GSEA analysis indicated that TAZ-, but not YAP-, regulated pathways [29] were significantly depleted upon *MESH1* knockdown in H1975 (Supplementary Fig. 3g). In the five indicated tumor datasets, tumors with low MESH1 expression levels also displayed low TAZ expression (Supplementary Fig. 3h), consistent with the regulatory relationships established in cultured cells. However, such a relationship was not found in other tumor datasets, which may be due to the various confounding environmental factors. Collectively, these data indicated that *MESH1* knockdown specifically repressed TAZ mRNA and pathway activity by a noncanonical mechanism.

**Fig. 3:**
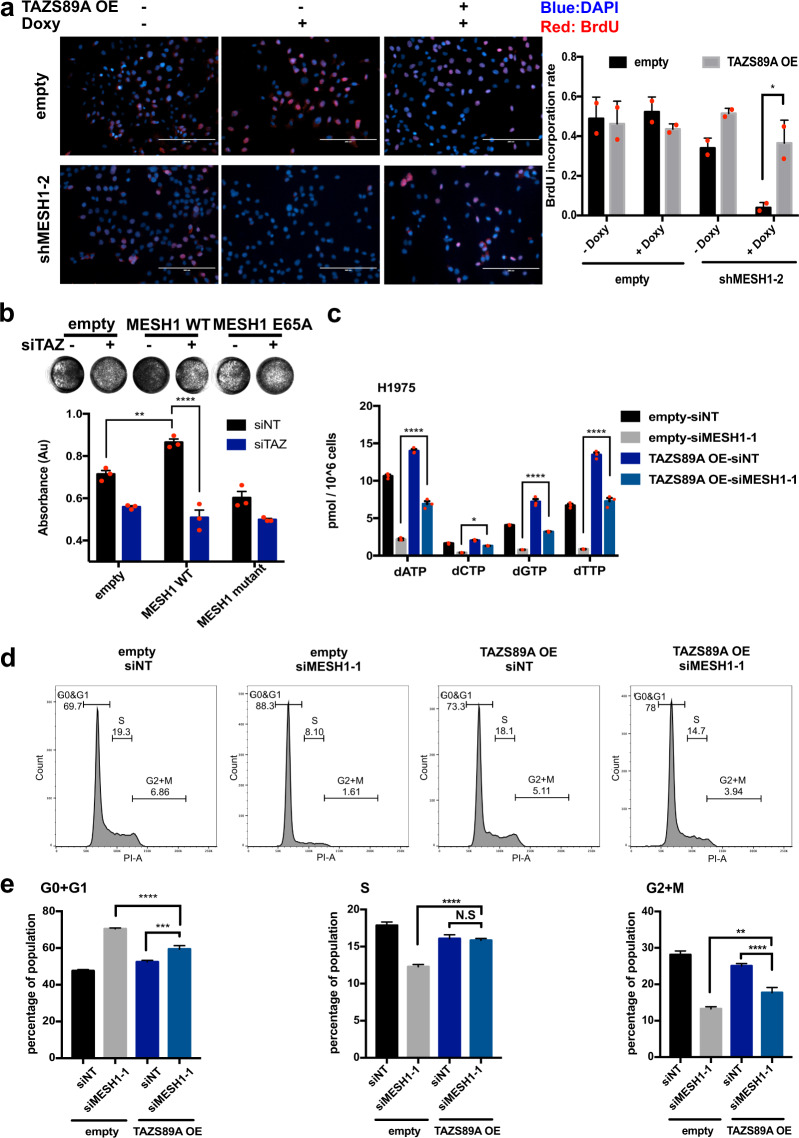

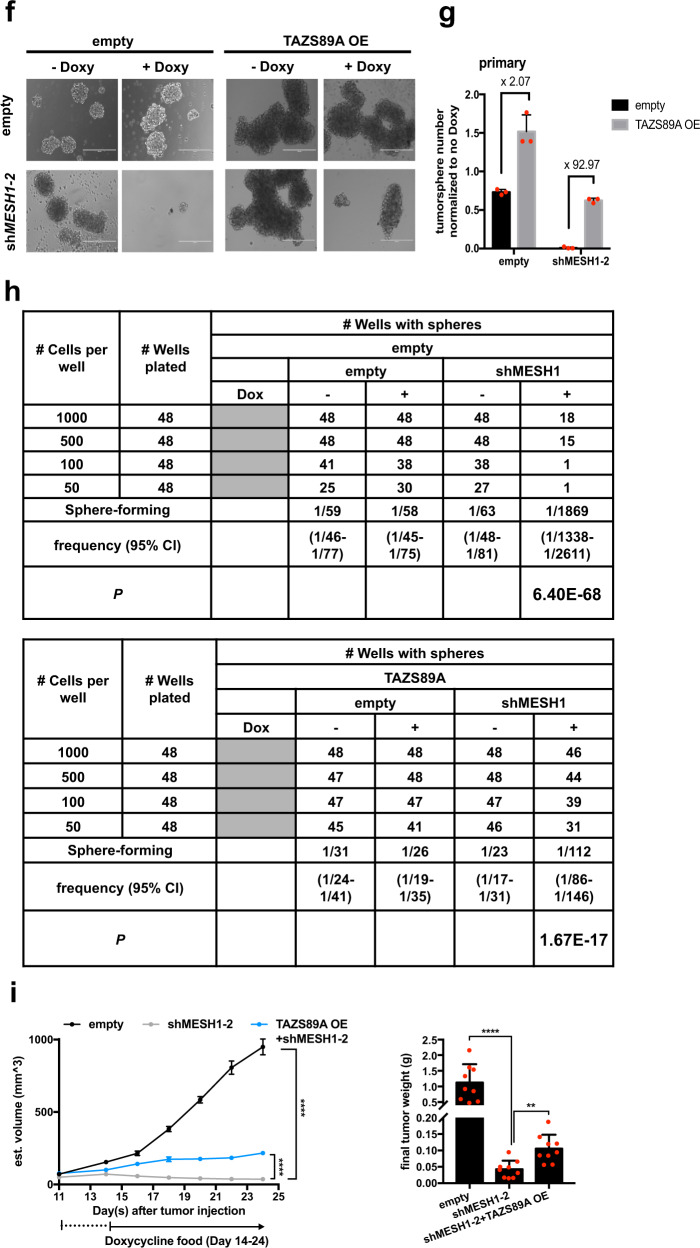
*TAZ* repression contributed significantly to the proliferation arrests of *MESH1* knockdown in H1975 cells. **a** Representative images (left) and quantification (right) of the BrdU incorporation assay associated with *MESH1* knockdown with or without the overexpression of TAZS89A. TAZS89A restoration significantly mitigated the inhibition of BrdU incorporation rate by *MESH1* knockdown. Scale bars: 200 μm. (mean + s.d.). **b** Representative image (top) of the crystal violet staining and quantification for replicates (bottom) of H1975 cells showed that *TAZ* removal abolished the enhanced cell growth by *MESH1* expression. (mean + s.d.). **c** dNTP measurement by MS-based analysis showed that TAZ restoration significantly mitigated the reduction of all four dNTPs by *MESH1* knockdown in H1975 cells. (mean + s.d.). **d** Cell cycle distribution by PI stain and **e** quantification (*n* = 3, mean + s.d.) of different cell cycle phases associated with *MESH1* knockdown with or without the overexpression of TAZS89A. The percentage of cells in each individual stage was calculated by the FlowJo software and labeled in the histogram. TAZS89A restoration significantly promoted cell cycle progression in *MESH1*-silenced cells arrested at the G0 + G1 stage. **f** Representative image and **g** quantification (mean + s.d.) of the primary tumor sphere assay with TAZS89A expression revealed that TAZS89A restoration upon *MESH1* knockdown increased tumor sphere numbers by ~92 folds in H1975. **h** Tumor sphere (stem cell) frequency by the limiting dilution assay of inducible *MESH1* knockdown and TAZS89A expression revealed that *TAZ* restoration significantly expanded the stem cell pool decreased by *MESH1* knockdown. **i** Tumor size and weight measurement showed the rescue of reduced xenograft growth of *MESH1* knockdown by TAZS89A expression. For **a**; **b**; **c**; **e**; **i**, *p* values were calculated by the two-way ANOVA followed by Tukey’s posttest. *P~(0.01, 0.05); **P~(0.001, 0.01); ***P~(0.0001, 0.001); ****P < 0.0001.

Next, the contribution of *TAZ* repression to various phenotypic alterations of *MESH1* knockdown was investigated by restoring the constitutively active TAZS89A [15, 30] (Supplementary Figs. 4a, 5a). TAZ restoration significantly rescued the reduced cell number (Supplementary Fig. 4b) and BrdU incorporation (Fig. 3a) upon *MESH1* knockdown. In contrast, *TAZ* depletion completely ablated the stimulation of cell growth by *MESH1* overexpression (Fig. 3b). Consistently, TAZS89A expression partially rescued the dNTP depletion (Fig. 3c) and cell cycle arrest by *MESH1* knockdown (Fig. 3d, e; gating strategy shown in Supplementary Fig. 4c).

TAZ is known to regulate the self-renewal capacity of cancer cells and tumor growth [23]. Indeed, TAZS89A increased the number of spheres by ~92 folds in the shMESH1 groups, whereas only ~2 folds in the control (Fig. 3f, g), a trend persisted in the secondary sphere formation assay (Supplementary Fig. 5b). Furthermore, the limiting dilution assay showed that *MESH1* knockdown massively reduced the stem cell frequency from 1/63 to 1/1869 in H1975, and was partially restored by TAZS89A expression to 1/112 (Fig. 3h). Next, we tested the potential of TAZ restoration in mitigating the tumor inhibition effects of *MESH1* knockdown in xenografts. We found that the in vivo expression of TAZS89A in the MESH1-removed xenografts rescued the tumor volume and weight by ~3 folds (Fig. 3i and Supplementary Fig. 5c). Collectively, these data indicated the importance of *TAZ* repression to the dNTP depletion, proliferation arrest, reduced sphere formation, and tumor growth inhibition phenotypes associated with *MESH1* knockdown. However, TAZ restoration only partially reversed these antitumor phenotypes of *MESH1* removal, indicating that TAZ repression may not be the only factor that contributed.

### *TAZ* downregulation contributed to the transcriptional changes of *MESH1* knockdown

**Fig. 4:**
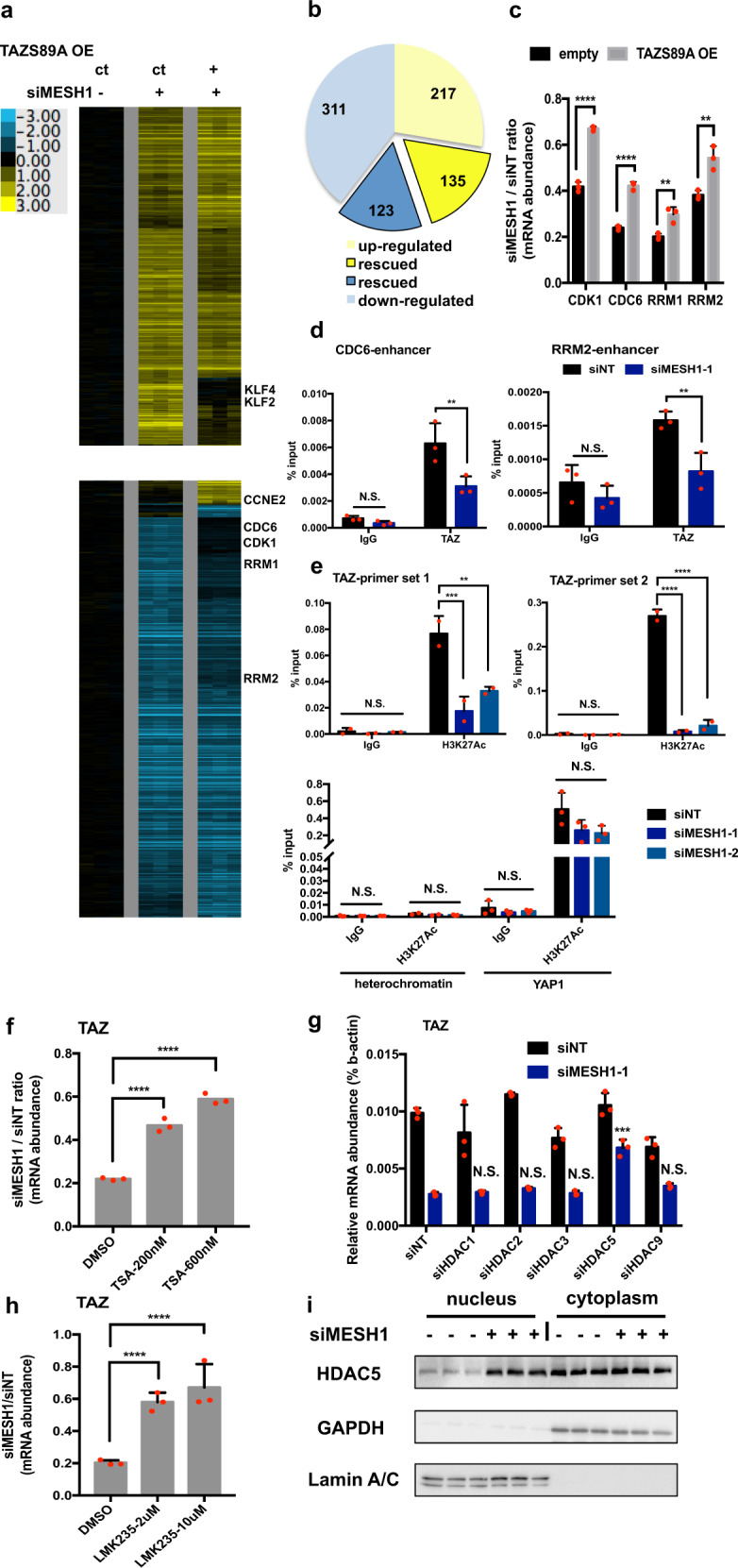
The contribution of *TAZ* repression to the transcriptional responses of *MESH1* knockdown in H1975 cells. **a** Heatmap of the selected genes in response to the *MESH1* knockdown with or without TAZS89A restoration. Filtering resulted in 1007 probesets: at least four observations with absolute values ≥0.7 were included. ct represents control. **b** Quantification of the *MESH1*-silenced gene signatures that were restored by TAZS89A restoration. 33% (258 out of 786 genes) were rescued. Expression changes ≥2 folds were included as the down or upregulated genes, among which the ones that were rescued by TAZS89A restoration by at least 1.5 folds were considered as “rescued”. **c** qRT-PCR validation of the selected genes in **a**. TAZS89A restoration significantly rescued the inhibition of cell cycle-related gene expression by *MESH1* knockdown. (mean + s.d.). **d** ChIP-qPCR analysis of the abundance of TAZ protein at the enhancer region of *CDC6* and *RRM2* in cells transfected with the control or *MESH1* siRNA. (mean + s.d.). **e** ChIP-qPCR analysis of the abundance of H3K27Ac mark at the promoter region of *TAZ*, *YAP*, and a heterochromatin region in cells transfected with siNT or two si*MESH1*s. (mean + s.d.). **f** qRT-PCR validation revealed that TSA treatment for 16 h rescued *TAZ* repression by *MESH1* knockdown. (mean + s.d.). **g** qRT-PCR revealed that only si*HDAC5* significantly rescued *TAZ* repression by si*MESH1*. (mean + s.d.). **h** qRT-PCR validation revealed that LMK235 treatment for 24 h rescued *TAZ* repression by *MESH1* knockdown. (mean + s.d.). **i** Representative image of the western blots showed an enhanced nucleus translocation of HDAC5 by *MESH1* knockdown, implying activation of HDAC5. For **c**, *p* values were calculated by the two-tail student’s *t*-test. For **d**; **e**; **g**, *p* values were calculated by the two-way ANOVA followed by Tukey’s posttest. For **f**; **h**, *p* values were calculated by the one-way ANOVA followed by Tukey’s posttest. **P~(0.001, 0.01); ***P~(0.0001, 0.001); ****P < 0.0001; NS no significance.

As TAZ functions as a transcription coactivator, we determined the role of *TAZ* repression in regulating the transcriptional responses to *MESH1* knockdown (Fig. 4) by comparing the *MESH1*-knockdown gene signatures between the control and TAZS89A-transfected groups (Fig. 4a). While the transcriptional responses were similar, *TAZ* restoration mitigated the *MESH1* knockdown-affected changes in ~33% (258 out of 786 genes) of both the repressed (123 of 434 genes) and induced genes (135 of 352 genes) (Fig. 4b), and the rescued gene lists were included in Table 2. Interestingly, the MESH1-affected genes restored by TAZ expression included several known cell cycle-related genes, such as *CDC6*, *CDK1*, *RRM1, RRM2* (Fig. 4c), *CCNE2* (Cyclin E2), *KLF2* (Kruppel-like factor 2), and *KLF4* (Kruppel-like factor 4) [25, 31, 32]. ChIP-qPCR further showed the binding of TAZ to the regulatory regions of *CDC6* and *RRM2* (Fig. 4d) as a transcriptional effector. Overall, these results indicated that *TAZ* repression mediated ~1/3 of the transcriptional response to *MESH1* knockdown, including the downregulation of many cell cycle-related genes that contributed to the anti-growth effects (Fig. 3).

**Table 2:** The list of MESH1-affected genes which was rescued by the expression of TAZS89A.

Gene title	Gene symbol	
**B-cell linker**	**BLNK**	
**aldo-keto reductase family 1, member C3 (3-alpha hydroxysteroid dehydrogenase, type II)**	**AKR1C3**	Note:Entries shown in bold denote the genes restored by TAZS89A expression by at least 1.5 folds
**chemokine (C-C motif) ligand 5**	**CCL5**	
**homeobox D1**	**HOXD1**	
**KIAA0125**	**KIAA0125**	
**V-set domain containing T cell activation inhibitor 1**	**VTCN1**	
**chemokine (C-C motif) ligand 5 /// chemokine (C-C motif) ligand 5**	**CCL5**	
**chitinase 3-like 1 (cartilage glycoprotein-39)**	**CHI3L1**	
**chitinase 3-like 1 (cartilage glycoprotein-39)**	**CHI3L1**	
**phosphodiesterase 4D interacting protein (myomegalin) /// similar to phosphodiesterase 4D interacting protein isoform 1**	**PDE4DIP /// LOC727893**	
**tumor necrosis factor (ligand) superfamily, member 10 /// tumor necrosis factor (ligand) superfamily, member 10**	**TNFSF10**	
**chloride intracellular channel 2**	**CLIC2**	
**SP100 nuclear antigen**	**SP100**	
**Chromosome 5 open reading frame 13**	**C5orf13**	
**meningioma (disrupted in balanced translocation) 1**	**MN1**	
**chromosome 10 open reading frame 81**	**C10orf81**	
**C-type lectin domain family 2, member B /// CMT1A duplicated region transcript 15 pseudogene**	**CLEC2B /// CDRT15P**	
**major histocompatibility complex, class II, DP alpha 1**	**HLA-DPA1**	
**nuclear factor (erythroid-derived 2), 45** **kDa**	**NFE2**	
**major histocompatibility complex, class II, DR alpha**	**HLA-DRA**	
**neuron navigator 3**	**NAV3**	
**SRY (sex determining region Y)-box 2**	**SOX2**	
**ATP-binding cassette, subfamily A (ABC1), member 1**	**ABCA1**	
**POU domain, class 2, transcription factor 3**	**POU2F3**	
**LY6/PLAUR domain containing 1**	**LYPD1**	
**secreted phosphoprotein 1 (osteopontin, bone sialoprotein I, early T-lymphocyte activation 1)**	**SPP1**	
**vav3 oncogene**	**VAV3**	
**guanine nucleotide binding protein (G protein), alpha activating activity polypeptide, olfactory type**	**GNAL**	
**guanylate binding protein 1, interferon-inducible, 67** **kDa /// guanylate binding protein 1, interferon-inducible, 67** **kDa**	**GBP1**	
**cathepsin S**	**CTSS**	
**phosphodiesterase 4D interacting protein (myomegalin)**	**PDE4DIP**	
**ATP-binding cassette, subfamily A (ABC1), member 1**	**ABCA1**	
**guanylate binding protein 1, interferon-inducible, 67** **kDa /// guanylate binding protein 1, interferon-inducible, 67** **kDa**	**GBP1**	
**zinc finger and BTB domain containing 1**	**ZBTB1**	
**major histocompatibility complex, class II, DM beta /// major histocompatibility complex, class II, DM beta**	**HLA-DMB**	
**major histocompatibility complex, class II, DR alpha /// major histocompatibility complex, class II, DR alpha**	**HLA-DRA**	
**tumor necrosis factor (ligand) superfamily, member 10 /// tumor necrosis factor (ligand) superfamily, member 10**	**TNFSF10**	
**major histocompatibility complex, class II, DM alpha**	**HLA-DMA**	
**microseminoprotein, beta-**	**MSMB**	
**XIAP associated factor-1**	**BIRC4BP**	
**phosphodiesterase 4D interacting protein (myomegalin)**	**PDE4DIP**	
**neuron navigator 2**	**NAV2**	
**LIM domain only 2 (rhombotin-like 1)**	**LMO2**	
**tight junction protein 3 (zona occludens 3)**	**TJP3**	
**cancer susceptibility candidate 1**	**CASC1**	
**chromosome 9 open reading frame 61**	**C9orf61**	
**hydroxyprostaglandin dehydrogenase 15-(NAD)**	**HPGD**	
**tight junction protein 3 (zona occludens 3)**	**TJP3**	
**cathepsin S**	**CTSS**	
**2′,5′-oligoadenylate synthetase 1, 40/46** **kDa**	**OAS1**	
**guanylate binding protein 2, interferon-inducible /// guanylate binding protein 2, interferon-inducible**	**GBP2**	
**matrix metallopeptidase 13 (collagenase 3) /// matrix metallopeptidase 13 (collagenase 3)**	**MMP13**	
**ATP-binding cassette, subfamily A (ABC1), member 12**	**ABCA12**	
**angiotensinogen (serpin peptidase inhibitor, clade A, member 8)**	**AGT**	
**Metallophosphoesterase 1**	**MPPE1**	
**cysteine-rich, angiogenic inducer, 61**	**CYR61**	
**kelch-like 24 (Drosophila)**	**KLHL24**	
**tumor necrosis factor (ligand) superfamily, member 10 /// tumor necrosis factor (ligand) superfamily, member 10**	**TNFSF10**	
**indoleamine-pyrrole 2,3 dioxygenase**	**INDO**	
**cysteine-rich, angiogenic inducer, 61**	**CYR61**	
**snail homolog 2 (Drosophila)**	**SNAI2**	
**bradykinin receptor B2**	**BDKRB2**	
**platelet-derived growth factor D**	**PDGFD**	
**killer cell lectin-like receptor subfamily C, member 3**	**KLRC3**	
**fatty acid 2-hydroxylase**	**FA2H**	
**GRAM domain containing 1** **C**	**GRAMD1C**	
**hydroxyprostaglandin dehydrogenase 15-(NAD)**	**HPGD**	
**S100 calcium-binding protein P**	**S100P**	
**mucin 16, cell surface-associated**	**MUC16**	
**cytidine monophosphate-N-acetylneuraminic acid hydroxylase (CMP-*N*-acetylneuraminate monooxygenase)**	**CMAH**	
**tumor protein p73-like**	**TP73L**	
**solute carrier family 28 (sodium-coupled nucleoside transporter), member 3**	**SLC28A3**	
**Immunoglobulin heavy constant alpha 1**	**IGHA1**	
**myxovirus (influenza virus) resistance 1, interferon-inducible protein p78 (mouse) /// myxovirus (influenza virus) resistance 1, interferon-inducible protein p78 (mouse)**	**MX1**	
**septin 4**	**4-Sep**	
**complement factor B**	**CFB**	
**v-maf musculoaponeurotic fibrosarcoma oncogene homolog (avian)**	**MAF**	
**LIM domain binding 3**	**LDB3**	
**hydroxyprostaglandin dehydrogenase 15-(NAD)**	**HPGD**	
**Metallophosphoesterase 1**	**MPPE1**	
**kelch-like 24 (Drosophila)**	**KLHL24**	
**2′,5′-oligoadenylate synthetase 1, 40/46** **kDa**	**OAS1**	
**interleukin 1 receptor, type I**	**IL1R1**	
**advillin**	**AVIL**	
**radical S-adenosyl methionine domain containing 2**	**RSAD2**	
**4-aminobutyrate aminotransferase**	**ABAT**	
**CCAAT/enhancer-binding protein (C/EBP), delta**	**CEBPD**	
**immunoglobulin heavy constant alpha 1 /// immunoglobulin heavy constant alpha 2 (A2m marker)**	**IGHA1 /// IGHA2**	
**Ral GEF with PH domain and SH3 binding motif 1**	**RALGPS1**	
**solute carrier family 16, member 4 (monocarboxylic acid transporter 5)**	**SLC16A4**	
**adrenergic, beta-1-, receptor**	**ADRB1**	
**catenin (cadherin-associated protein), alpha 2**	**CTNNA2**	
**SLAM family member 7**	**SLAMF7**	
**Kruppel-like factor 4 (gut)**	**KLF4**	
***N*-acylsphingosine amidohydrolase (acid ceramidase) 1**	**ASAH1**	
**phosphodiesterase 4D interacting protein (myomegalin)**	**PDE4DIP**	
**vacuolar protein sorting 13 homolog C (S. cerevisiae)**	**VPS13C**	
**GABA(A) receptor-associated protein like 1 /// GABA(A) receptors associated protein like 3**	**GABARAPL1 /// GABARAPL3**	
**Kruppel-like factor 4 (gut)**	**KLF4**	
**connective tissue growth factor**	**CTGF**	
**RAB15, member RAS onocogene family**	**RAB15**	
**desmocollin 2**	**DSC2**	
**chromosome 5 open reading frame 13**	**C5orf13**	
**histone cluster 1, H4h**	**HIST1H4H**	
**interferon-stimulated transcription factor 3, gamma 48** **kDa**	**ISGF3G**	
**histone cluster 1, H2am**	**HIST1H2AM**	
**histone cluster 1, H2ae**	**HIST1H2AE**	
**HGFL gene /// HGFL gene**	**MGC17330**	
**myeloid/lymphoid or mixed-lineage leukemia (trithorax homolog, Drosophila); translocated to, 3**	**MLLT3**	
**Trophoblast-derived noncoding RNA**	**TncRNA**	
**hect domain and RLD 6**	**HERC6**	
**pre-B-cell leukemia transcription factor interacting protein 1**	**PBXIP1**	
**histone cluster 1, H2ag**	**HIST1H2AG**	
**similar to myeloid/lymphoid or mixed-lineage leukemia (trithorax homolog, Drosophila); translocated to, 4**	**LOC653483**	
---	---	
**solute carrier family 2 (facilitated glucose/fructose transporter), member 5**	**SLC2A5**	
**solute carrier family 12 (potassium/chloride transporters), member 8**	**SLC12A8**	
**Kruppel-like factor 2 (lung)**	**KLF2**	
**chromosome 5 open reading frame 13**	**C5orf13**	
**melanoma inhibitory activity family, member 3**	**MIA3**	
**serine/threonine kinase 38 like**	**STK38L**	
**LAG1 homolog, ceramide synthase 4 (S. cerevisiae)**	**LASS4**	
**thioredoxin interacting protein**	**TXNIP**	
**nuclear protein 1**	**NUPR1**	
**histone deacetylase 9**	**HDAC9**	
**GABA(A) receptor-associated protein like 1**	**GABARAPL1**	
**pre-B-cell leukemia transcription factor interacting protein 1**	**PBXIP1**	
***N*-acylsphingosine amidohydrolase (acid ceramidase) 1**	**ASAH1**	
**interferon-induced protein 44-like**	**IFI44L**	
**glutamate-ammonia ligase (glutamine synthetase)**	**GLUL**	
**fibroblast growth factor receptor 3 (achondroplasia, thanatophoric dwarfism)**	**FGFR3**	
**Full-length cDNA clone CS0DK002YF13 of HeLa cells Cot 25-normalized of Homo sapiens (human)**	**---**	
**reelin**	**RELN**	
**cadherin 5, type 2, VE-cadherin (vascular epithelium)**	**CDH5**	
**phosphatidic acid phosphatase type 2A**	**PPAP2A**	
WD repeat domain 19	WDR19	
thioredoxin interacting protein	TXNIP	
myeloid/lymphoid or mixed-lineage leukemia (trithorax homolog, Drosophila); translocated to, 3	MLLT3	
tripeptidyl peptidase I	TPP1	
enhancer of zeste homolog 1 (Drosophila)	EZH1	
reticulon 2	RTN2	
chromosome 9 open reading frame 95	C9orf95	
frizzled homolog 5 (Drosophila) /// frizzled homolog 5 (Drosophila)	FZD5	
histone cluster 2, H2be	HIST2H2BE	
dedicator of cytokinesis 4	DOCK4	
damage-regulated autophagy modulator	DRAM	
dynamin 3	DNM3	
glutamyl aminopeptidase (aminopeptidase A)	ENPEP	
histone cluster 1, H2ac	HIST1H2AC	
spermidine/spermine N1-acetyltransferase 1	SAT1	
thioredoxin interacting protein	TXNIP	
desmocollin 2	DSC2	
protease, serine, 8 (prostasin)	PRSS8	
inositol 1,4,5-triphosphate receptor, type 2	ITPR2	
secreted protein, acidic, cysteine-rich (osteonectin) /// secreted protein, acidic, cysteine-rich (osteonectin)	SPARC	
plexin A2	PLXNA2	
RB1-inducible coiled-coil 1	RB1CC1	
argininosuccinate synthetase 1	ASS1	
spermidine/spermine N1-acetyltransferase 1	SAT1	
solute carrier family 35, member D2	SLC35D2	
yippee-like 5 (Drosophila)	YPEL5	
phosphatidic acid phosphatase type 2A	PPAP2A	
myosin, light chain 9, regulatory	MYL9	
major histocompatibility complex, class I, E	HLA-E	
zinc finger protein 91	ZNF91	
cathepsin O	CTSO	
GULP, engulfment adapter PTB domain containing 1	GULP1	
solute carrier family 35, member D2	SLC35D2	
clusterin	CLU	
brain expressed, associated with Nedd4	BEAN	
BTB and CNC homology 1, basic leucine zipper transcription factor 1	BACH1	
programmed cell death 4 (neoplastic transformation inhibitor)	PDCD4	
B-cell CLL/lymphoma 6 (zinc finger protein 51)	BCL6	
spermidine/spermine *N*1-acetyltransferase 1	SAT1	
secreted protein, acidic, cysteine-rich (osteonectin)	SPARC	
granulin	GRN	
Kruppel-like factor 7 (ubiquitous)	KLF7	
granulin	GRN	
granulin	GRN	
solute carrier family 9 (sodium/hydrogen exchanger), member 6	SLC9A6	
sperm associated antigen 9	SPAG9	
pleiomorphic adenoma gene 1	PLAG1	
vav3 oncogene	VAV3	
synaptotagmin I	SYT1	
KIAA0323	KIAA0323	
hypothetical protein FLJ20054	FLJ20054	
growth arrest and DNA-damage-inducible, beta	GADD45B	
B-cell CLL/lymphoma 6 (zinc finger protein 51) /// B-cell CLL/lymphoma 6 (zinc finger protein 51)	BCL6	
microtubule-associated protein 1 light chain 3 gamma /// microtubule-associated protein 1 light chain 3 gamma	MAP1LC3C	
chromosome 14 open reading frame 45	C14orf45	
neural precursor cell expressed, developmentally downregulated 4	NEDD4	
---	---	
growth arrest and DNA-damage-inducible, beta	GADD45B	
dimethylarginine dimethylaminohydrolase 1	DDAH1	
Pre-B-cell leukemia transcription factor 1	PBX1	
WD repeat domain 78	WDR78	
plasminogen-like B2 /// plasminogen-like B1	PLGLB2 /// PLGLB1	
histone cluster 1, H2bi	HIST1H2BI	
cysteine-rich protein 2	CRIP2	
histone cluster 1, H2bg /// histone cluster 1, H2bc	HIST1H2BG /// HIST1H2BC	
amyotrophic lateral sclerosis 2 (juvenile) chromosome region, candidate 8	ALS2CR8	
histone cluster 2, H2aa3 /// histone cluster 2, H2aa4	HIST2H2AA3 /// HIST2H2AA4	
NIMA (never in mitosis gene a)-related kinase 7	NEK7	
cut-like 1, CCAAT displacement protein (Drosophila)	CUTL1	
clusterin	CLU	
MORC family CW-type zinc finger 3	MORC3	
histone cluster 1, H2bf	HIST1H2BF	
type 1 tumor necrosis factor receptor shedding aminopeptidase regulator	ARTS-1	
phosphoinositide-3-kinase, regulatory subunit 3 (p55, gamma)	PIK3R3	
histone cluster 1, H2bk	HIST1H2BK	
epidermal growth factor receptor pathway substrate 15	EPS15	
chromosome 1 open reading frame 107	C1orf107	
BMP2 inducible kinase	BMP2K	
histone cluster 2, H2aa3 /// histone cluster 2, H2aa4	HIST2H2AA3 /// HIST2H2AA4	
histone cluster 1, H2be	HIST1H2BE	
histone cluster 1, H2bh	HIST1H2BH	
mitochondrial tumor suppressor 1	MTUS1	
transducin-like enhancer of split 1 (E(sp1) homolog, Drosophila)	TLE1	
solute carrier family 17 (anion/sugar transporter), member 5	SLC17A5	
stomatin	STOM	
similar to phosphodiesterase 4D interacting protein isoform 2	LOC727942	
chondroitin sulfate GalNAcT-2	GALNACT-2	
---	---	
dual-specificity tyrosine-(Y)-phosphorylation regulated kinase 2	DYRK2	
son of sevenless homolog 2 (Drosophila)	SOS2	
centrosomal protein 68 kDa	CEP68	
Tudor domain containing 7	TDRD7	
mRNA; cDNA DKFZp667B0924 (from clone DKFZp667B0924)	---	
ras homolog gene family, member Q	RHOQ	
KIAA0831	KIAA0831	
mitochondrial tumor suppressor 1	MTUS1	
chromosome 11 open reading frame 63	C11orf63	
zinc finger protein 467	ZNF467	
Cas-Br-M (murine) ecotropic retroviral transforming sequence b	CBLB	
protein tyrosine phosphatase type IVA, member 1	PTP4A1	
MADS box transcription enhancer factor 2, polypeptide C (myocyte enhancer factor 2 C)	MEF2C	
suppressor of cytokine signaling 5	SOCS5	
testis-specific, 10	TSGA10	
CDC42 effector protein (Rho GTPase binding) 3	CDC42EP3	
Ras association (RalGDS/AF-6) domain family 3	RASSF3	
creatine kinase, mitochondrial 1B /// creatine kinase, mitochondrial 1 A	CKMT1B /// CKMT1A	
selenium binding protein 1 /// selenium binding protein 1	SELENBP1	
retinitis pigmentosa 2 (X-linked recessive)	RP2	
hypothetical protein MGC24039	MGC24039	
SEC24 related gene family, member A (S. cerevisiae)	SEC24A	
unc-51-like kinase 1 (C. elegans)	ULK1	
alkaline phosphatase, placental-like 2	ALPPL2	
---	---	
annexin A4	ANXA4	
interferon regulatory factor 7	IRF7	
interferon, alpha-inducible protein 27	IFI27	
Clone 23548 mRNA sequence	---	
---	---	
poly(A) binding protein-interacting protein 1	PAIP1	
spectrin repeat containing, nuclear envelope 2	SYNE2	
testis-specific kinase 2	TESK2	
transducin-like enhancer of split 1 (E(sp1) homolog, Drosophila)	TLE1	
cDNA FLJ31107 fis, clone IMR322000152	---	
dpy-19-like 1 (C. elegans)	DPY19L1	
histone cluster 1, H3h	HIST1H3H	
SH3-domain GRB2-like endophilin B1	SH3GLB1	
checkpoint suppressor 1	CHES1	
GRIP and coiled-coil domain containing 2	GCC2	
dipeptidyl-peptidase 4 (CD26, adenosine deaminase complexing protein 2)	DPP4	
histone cluster 1, H2bd	HIST1H2BD	
Ribosomal protein L41	RPL41	
zinc finger, FYVE domain containing 26	ZFYVE26	
dipeptidyl-peptidase 4 (CD26, adenosine deaminase complexing protein 2)	DPP4	
Activating transcription factor 6	ATF6	
zinc finger, FYVE domain containing 26	ZFYVE26	
vacuolar protein sorting 4 homolog B (S. cerevisiae)	VPS4B	
programmed cell death 4 (neoplastic transformation inhibitor)	PDCD4	
suppressor of cytokine signaling 5	SOCS5	
checkpoint suppressor 1	CHES1	
son of sevenless homolog 2 (Drosophila) /// son of sevenless homolog 2 (Drosophila)	SOS2	
chloride intracellular channel 3 /// rabaptin, RAB GTPase binding effector protein 1	CLIC3 /// RABEP1	
ankyrin repeat domain 46	ANKRD46	
serum/glucocorticoid regulated kinase family, member 3	SGK3	
histone cluster 1, H3d	HIST1H3D	
phosphodiesterase 4D, cAMP-specific (phosphodiesterase E3 dunce homolog, Drosophila)	PDE4D	
cylindromatosis (turban tumor syndrome)	CYLD	
protocadherin alpha 9 /// protocadherin alpha subfamily C, 2 /// protocadherin alpha subfamily C, 1 /// protocadherin alpha 13 /// protocadherin alpha 12 /// protocadherin alpha 11 /// protocadherin alpha 10 /// protocadherin alpha 8 /// protocadherin alpha 7 /// protocadherin alpha 6 /// protocadherin alpha 5 /// protocadherin alpha 4 /// protocadherin alpha 3 /// protocadherin alpha 2 /// protocadherin alpha 1	PCDHA9 /// PCDHAC2 /// PCDHAC1 /// PCDHA13 /// PCDHA12 /// PCDHA11 /// PCDHA10 /// PCDHA8 /// PCDHA7 /// PCDHA6 /// PCDHA5 /// PCDHA4 /// PCDHA3 /// PCDHA2 /// PCDHA1	
programmed cell death 4 (neoplastic transformation inhibitor)	PDCD4	
solute carrier family 35 (UDP-*N*-acetylglucosamine (UDP-GlcNAc) transporter), member A3	SLC35A3	
family with sequence similarity 59, member A	FAM59A	
platelet-activating factor acetylhydrolase, isoform Ib, alpha subunit 45 kDa	PAFAH1B1	
chondroitin sulfate GalNAcT-2	GALNACT-2	
interleukin 7 receptor /// interleukin 7 receptor	IL7R	
Chromosome 20 open reading frame 111	C20orf111	
chromosome 14 open reading frame 101	C14orf101	
E74-like factor 3 (ETS-domain transcription factor, epithelial-specific)	ELF3	
leucine zipper transcription factor-like 1	LZTFL1	
aquaporin 3 (Gill blood group)	AQP3	
ring finger and KH domain containing 2	RKHD2	
programmed cell death 4 (neoplastic transformation inhibitor)	PDCD4	
KIAA0329	KIAA0329	
nuclear receptor coactivator 1	NCOA1	
zinc finger, CCHC domain containing 14	ZCCHC14	
TSC22 domain family, member 3	TSC22D3	
inhibitor of DNA binding 2, dominant negative helix-loop-helix protein /// inhibitor of DNA binding 2B, dominant negative helix-loop-helix protein	ID2 /// ID2B	
kelch repeat and BTB (POZ) domain containing 10	KBTBD10	
pregnancy specific beta-1-glycoprotein 5	PSG5	
tuftelin 1	TUFT1	
abhydrolase domain containing 5	ABHD5	
glycoprotein (transmembrane) nmb	GPNMB	
Rho GTPase activating protein 5	ARHGAP5	
inhibitor of DNA binding 2, dominant negative helix-loop-helix protein	ID2	
RANBP2-like and GRIP domain containing 5 /// RANBP2-like and GRIP domain containing 4 /// RANBP2-like and GRIP domain containing 8 /// RANBP2-like and GRIP domain containing 6	RGPD5 /// RGPD4 /// RGPD8 /// RGPD6	
Homo sapiens, clone IMAGE:4214654, mRNA	---	
fragile X mental retardation 1	FMR1	
fragile X mental retardation 1	FMR1	
lysine-rich coiled-coil 1	KRCC1	
deiodinase, iodothyronine, type II	DIO2	
calmodulin regulated spectrin-associated protein 1-like 1	CAMSAP1L1	
syntaxin 6	STX6	
Immunoglobulin heavy constant alpha 1	IGHA1	
carboxypeptidase E	CPE	
calmodulin regulated spectrin-associated protein 1-like 1	CAMSAP1L1	
MADS box transcription enhancer factor 2, polypeptide C (myocyte enhancer factor 2 C)	MEF2C	
Schwannomin interacting protein 1	SCHIP1	
Dmx-like 1	DMXL1	
abhydrolase domain containing 5	ABHD5	
Homo sapiens, clone IMAGE:4214654, mRNA	---	
sphingomyelin phosphodiesterase, acid-like 3 A	SMPDL3A	
caveolin 2	CAV2	
FLJ20160 protein	FLJ20160	
pleckstrin homology domain containing, family C (with FERM domain) member 1	PLEKHC1	
pregnancy specific beta-1-glycoprotein 7	PSG7	
pregnancy specific beta-1-glycoprotein 4	PSG4	
pregnancy specific beta-1-glycoprotein 1	PSG1	
receptor accessory protein 5	REEP5	
RAB11 family interacting protein 1 (class I)	RAB11FIP1	
coiled-coil domain containing 92	CCDC92	
carboxypeptidase E	CPE	
hypothetical protein FLJ10357	FLJ10357	
piccolo (presynaptic cytomatrix protein)	PCLO	
dipeptidyl-peptidase 4 (CD26, adenosine deaminase complexing protein 2)	DPP4	
pregnancy specific beta-1-glycoprotein 9	PSG9	
HEG homolog 1 (zebrafish)	HEG1	
filamin A interacting protein 1-like	FILIP1L	
cyclin G2	CCNG2	
pregnancy specific beta-1-glycoprotein 6	PSG6	
inositol polyphosphate-5-phosphatase, 40 kDa	INPP5A	
pregnancy specific beta-1-glycoprotein 9	PSG9	
piccolo (presynaptic cytomatrix protein)	PCLO	
cyclin G2	CCNG2	
cyclin G2	CCNG2	
aquaporin 3 (Gill blood group)	AQP3	
C-type lectin domain family 7, member A /// C-type lectin domain family 7, member A	CLEC7A	
neural precursor cell expressed, developmentally downregulated 9	NEDD9	
HEG homolog 1 (zebrafish)	HEG1	

Next, we investigated the epigenetic modulations on TAZ measuring H3K27ac (canonical active enhancer mark) levels at TAZ promoter/enhancer regions annotated by the GeneHancer [33] (Supplementary Fig. 6a). *MESH1* knockdown significantly reduced the abundance of H3K27Ac at two separate loci at the promoter/enhancer region 2 of TAZ (Fig. 4e), suggesting a closed structure of the TAZ chromatin. Comparatively, H3K27Ac at the YAP promoter or the heterochromatin region was not significantly affected. Furthermore, *NADK* knockdown reversed the histone hypo-acetylation at TAZ promoter/enhancer region 2 (Supplementary Fig. 6b), implicating a key role of MESH1 enzymatic activity in employing its epigenetic modulation on TAZ. Importantly, epigenetic activation of TAZ by the CRISPR activation (CRISPRa) (Supplementary Fig. 6c), which recruited a transcriptional activation domain by two individual sgRNAs (designed by the SAM Cas9 activator design tool [34]) to TAZ promoter/enhancer region 2, also rescued the repressed expression of several TAZ target genes (Supplementary Fig. 6d, e). Finally, TAZ re-activation significantly mitigated the proliferation arrest (Supplemental Fig. 6f) upon *MESH1* knockdown. Altogether, these data revealed the critical role of TAZ epigenetic repression in inhibiting TAZ downstream gene expression and proliferation phenotypes of *MESH1* knockdown.

Histone deacetylases (HDACs) are known to reduce histone acetylation and form inhibitory complexes to repress gene expression [35]. Interestingly, *MESH1* knockdown induced *HDAC5* and *HDAC9* expression (Supplementary Fig. 7a, b), which might contribute to the *TAZ* repression. Indeed, the pan-HDAC inhibitor (Trichostatin A or TSA) reversed *TAZ* repression upon *MESH1* knockdown (Fig. 4f). To identify the specific HDAC(s) involved in TAZ regulation, we knocked down HDAC1, 2, 3, 5, and 9 individually and measured their effects on *TAZ* repression upon *MESH1* knockdown. Among all tested HDACs, only *HDAC5* knockdown rescued *TAZ* repression (Fig. 4g), consistent with the rescued effect of the HDAC5-specific inhibitor LMK235 (Fig. 4h). Since HDAC5 activity can be regulated by nuclear translocation [36], we fractionalized the nucleus vs. cytosol and found that *MESH1* knockdown also dramatically increased the nuclear fraction (putative transcriptionally active) of HDAC5 (Fig. 4i). Together, these data indicated that the increased levels and activities of HDAC5 by *MESH1* knockdown contributed to the *TAZ* repression.

### HDAC5 and AHRR formed a repressing complex to inhibit *TAZ* transcription

Here, we aim to identify the HDAC5-interacting transcription factor(s) that regulates TAZ expression. Toward this goal, we compared the list of 48 published HDAC5 interactors (BioGRID database: [37]) with all eight top documented TAZ-binding transcription factors (QIAGEN CLC Genomics Workbench [38]). Such a comparison identified AHRR (aryl-hydrocarbon receptor repressor) and AHR (aryl-hydrocarbon receptor) as potential candidates. AHRR was induced by *MESH1* knockdown (Supplementary Fig. 7c) and reported to be associated with HDAC5 as a repressing complex component [39] that competes with and inhibits the function of AHR [39]. AHR is predicted to target the regulatory regions of TAZ by QIAGEN CLC Genomics Workbench [38]. Therefore, we sought to determine the potential of AHRR and AHR in the regulation of TAZ by MESH1.

**Fig. 5:**
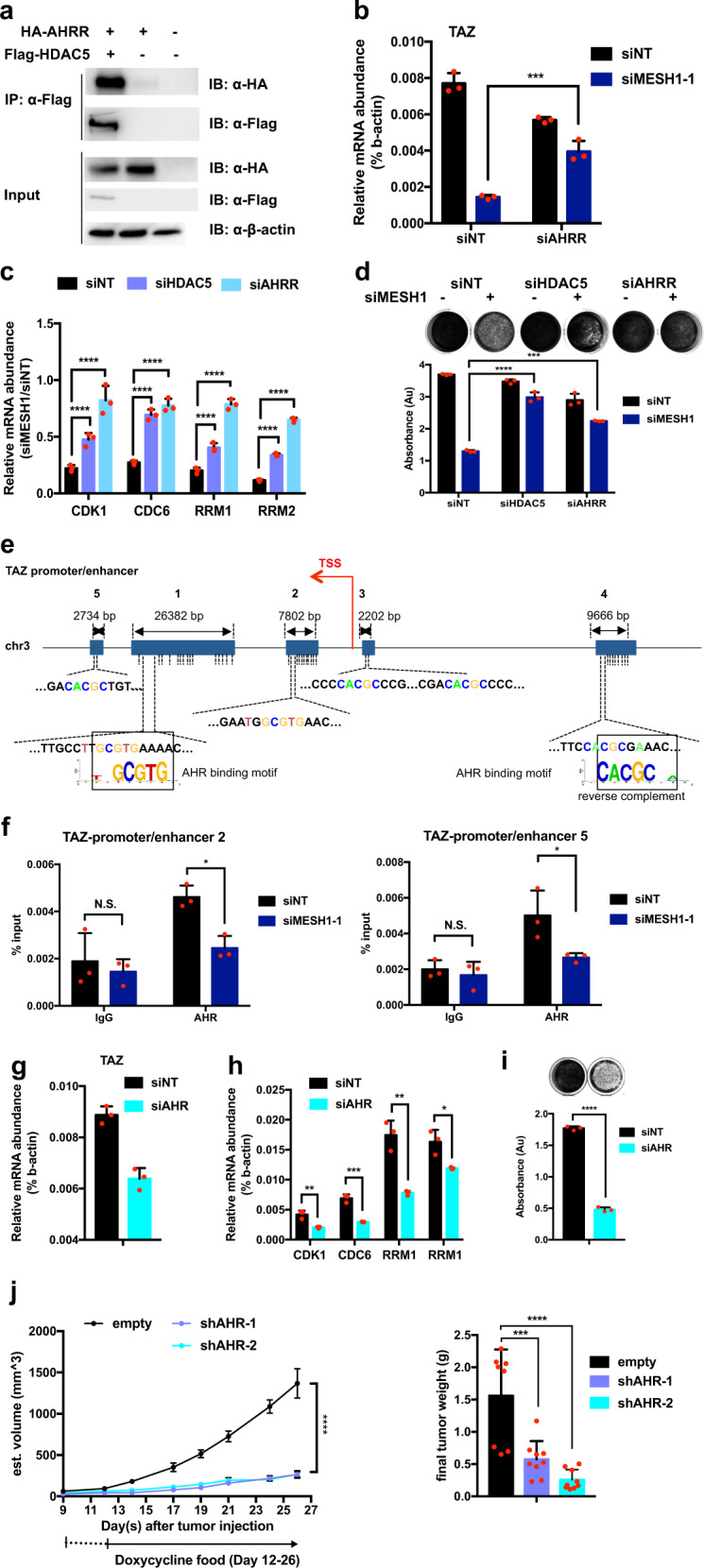
The effect of HDAC5 and AHRR as epigenetic co-repressors on *TAZ* downregulation and cell proliferation arrest in H1975. **a** Representative image of the co-immunoprecipitation and western blots suggested that HDAC5 interacted with AHRR in H1975 cells. The flag-HDAC5 and HA-AHRR were co-transfected into cells and flag-HDAC5 was immunoprecipitated by the flag antibody and probed with antibodies recognizing flag or HA tags. **b** qRT-PCR revealed that *AHRR* knockdown significantly rescued *TAZ* repression. (mean + s.d.). **c** qRT-PCR validation of the selected cell proliferation gene (*CDK1*, *CDC6*, *RRM1*, and *RRM2*) was rescued either by *AHRR* or *HDAC5* knockdown. (mean + s.d.). **d** Representative image of the crystal violet staining (top) and quantification for replicates (bottom) of H1975 cells showed a consistent resume of cell growth by *AHRR* and *HDAC5* knockdown. (mean + s.d.). **e** Analysis of binding motifs of AHR on the five TAZ promoter/enhancer regions identified by GeneHencer. AHR (TF) has several binding sites on multiple TAZ promoter/enhancer regions. Each dashed line represents a binding site and the transcription start site (TSS) of TAZ is marked with a red arrow. **f** AHR ChIP-qPCR data in H1975 showed enrichment at the TAZ promoter/enhancer region 2 and 5, which was repressed by *MESH1* knockdown. Cells were treated with ITE for 18 h to activate the AHR before ChIP assays. (mean + s.d.). **g** qRT-PCR validation of *TAZ* repression by *AHR* knockdown, consistent with the effect of *MESH1* knockdown. (mean + s.d.). **h** qRT-PCR validation of downregulation of the selected cell proliferation genes by *AHR* knockdown. (mean + s.d.). **i** Representative image of the crystal violet staining (top) and quantification for replicates (bottom) of H1975 cells showed inhibition of cell growth by *AHR* silencing. (mean + s.d.). **j** Tumor size and weight measurement showed xenograft growth inhibition upon doxycycline-induced *AHR* knockdown in the xenografted tumor model. *p* values were calculated by the one-way ANOVA followed by Tukey’s posttest. For **b**; **d**; **f**, *p* values were calculated by the two-way ANOVA followed by Tukey’s posttest. For **c**; **h**; **i**, *p* values were calculated by the two-tail student’s *t*-test. *P~(0.01, 0.05); **P~(0.001, 0.01); ***P~(0.0001, 0.001); ****P < 0.0001.

First, we confirmed that the restoration of wild-type, but not mutant, MESH1 abolished *HDAC5* and *AHRR* induction (Supplementary Fig. 7d). Next, co-knockdown of *NADK* and *MESH1* also diminished the induction of *HDAC5* and *AHRR* (Supplementary Fig. 7e), suggesting the relevance of MESH1’s enzymatic activity. Second, we validated the interaction between HDAC5 and AHRR by co-immunoprecipitation in H1975 cells transfected with HA-tagged AHRR and Flag-tagged HDAC5 (Fig. 5a). Third, *AHRR* knockdown (Supplementary Fig. 7c) substantially rescued *TAZ* repression by *MESH1* knockdown (Fig. 5b). Consistently, the knockdown of either *HDAC5* or *AHRR* increased the expression of *CDK1*, *CDK6*, *RRM1*, and *RRM2* (Fig. 5c) and the cell number (Fig. 5d). Overall, these data indicated that *MESHI1* knockdown upregulated *HDAC5* and *AHRR*, forming a repressing complex, to inhibit the expression of TAZ and its target genes, leading to the proliferation arrest.

Since AHR has been reported to bind at TAZ regulatory regions and promote TAZ transcription [26, 29], we investigated the potential that HDAC5 and AHRR could repress TAZ expression by inhibiting the binding of AHR to TAZ regulatory regions. The canonical AHR-responsive element (AHRE) contains the core sequence 5′-GCGTG-3′. Among the predicted AHRE sites in the five TAZ promoter/enhancer regions (Fig. 5e, annotated by the GeneHancer [33]), two were validated by ChIP-qPCR (Fig. 5f). More importantly, *MESH1* knockdown reduced the AHR occupancy at TAZ regulatory regions (Fig. 5f), consistent with the *TAZ* repression. Furthermore, *AHR* knockdown (Supplementary Fig. 7g), similar to *MESH1* knockdown, repressed *TAZ* (Fig. 5g) and proliferative gene expression (Fig. 5h) and inhibited cell proliferation (Fig. 5i) and tumor growth in vivo (Fig. 5j and Supplementary Fig. 7h, i). Importantly, tumors in the indicated four datasets with lower MESH1 expression have higher levels of HDAC5 (Supplementary Fig. 7j) in patients. In the tumor RNA-seq dataset that contained AHRR expression levels, tumors with low MESH1 expression also displayed a high level of AHRR (Supplementary Fig. 7k). Altogether, the abovementioned evidence supported the idea that *HDAC5* and *AHRR* upregulation upon *MESH1* knockdown mediated the proliferation arrest phenotypes by reducing AHR binding and TAZ transcription. In conclusion, we proposed a model by which *MESH1* knockdown exerts antitumor effects through repressing *TAZ* mRNA expression via an epigenetic modulation achieved by *HDAC5*-*AHRR* upregulation (Supplementary Fig. 8).

## Discussion

While metazoan genomes encode MESH1, the homolog of bacterial SpoT, our understanding of its functional role remains limited. Here, we have shown that *MESH1* knockdown triggered reproducible proliferation arrest and altered transcriptional patterns that mimicked the reduced proliferation phenotype of bacterial stringent response. First, the cell cycle arrest was associated with the repression of cell cycle gene expression, similar to the transcriptional feature of the stringent response [20]. Second, *MESH1* knockdown depleted dNTPs, reminiscent of the bacterial ppGpp-mediated dNTP depletion [11, 12]. However, *MESH1* knockdown also carried some distinct phenotypes, including *TAZ* repression via epigenetic modulations mediated by the induction of HDAC5/AHRR repressor complex. Therefore, our studies presented an interesting example in which the same protein homologs mediated similar phenotypes in different organisms via re-wiring through distinct substrates and mechanisms.

The Hippo signaling pathway exerts profound effects on cellular proliferation, survival, cell death, and organ sizes [25–28, 40, 41]. YAP and TAZ, two Hippo effectors, are usually tightly co-regulated by the phosphorylation of the kinase cascade of MST1/2, LATS1/2, and RASSF family proteins. Hence, our findings discovered a novel and interesting features. First, only TAZ, but not YAP, was affected by *MESH1* silencing. In fact, the YAP level was slightly increased, which might reflect a negative feedback loop of HIPPO activity noted in previous studies [42, 43]. Second, TAZ repression occurred at the mRNA instead of the posttranslational level. Previously, ETS (E26 transformation-specific) and MRTF/SRF have been reported to be involved in the transcriptional activation of *TAZ* mRNA [44, 45]. These results implied that MESH1 was also required to maintain the *TAZ* mRNA expression, the activity of the HIPPO pathway, and thus cellular proliferation. Interestingly, many components of the HIPPO pathway (including YAP and TAZ) first emerge in cnidarians, a very ancient group of metazoans [46]. All major domains of YAP and TAZ are also conserved between cnidarians and mammals. Given that MESH1 also shares the conserved domains with the bacterial hydrolase SpoT, it is tempting to speculate the functional convergence between MESH1 and the HIPPO pathway as they both appear in metazoans during evolution.

MESH1 was a cytosolic NADPH phosphatase [14]. Here, we discovered that the enzymatic activity is essential for the *HDAC5-AHRR* upregulation, *TAZ* repression, and antitumor effects. However, the underlying mechanisms are still unknown. In the future, it will be critical to investigate the connection between NADPH and transcriptional control of the epigenetic repressing complex. Furthermore, in bacteria, different environmental stresses (i.e., amino acid deprivation, heat shock, iron deficiency, etc.) trigger the ppGpp accumulation. While *MESH1* depletion leads to a stringent-like stress response, it is unclear which external stimuli activate the stringent response in metazoan, which requires further investigation. Despite these knowledge gaps, supported by the extensive amount of data, we demonstrated that MESH1 inhibition potentially represented a novel anti-growth response in cancer cells with significant therapeutic potentials. We can take advantage of the evolutionarily conserved pathways, proteins, and metabolites as well as the available X-ray structures of MESH1 [13, 14], to design MESH1 inhibitors and repress tumor proliferation as novel therapeutics.

## Materials and methods

### *MESH1*-knockdown using RNAi and shRNAs

Nontargeting siRNA (siNT) was purchased from Qiagen (AllStars Negative Control siRNA, SI03650318). *MESH1* targeted siRNAs were purchased from: Dharmacon: siMESH1-1 (target sequence GGGAAUCACUGACAUUGUG, D-031786-01); siMESH1-3 (target sequence GGACAGGAUUCAUACGCCA, J-031786-10); siNADK (target sequence UGAAUGAGGUGGUGAUUGA, CGCCAGCGAUGAAAGCUUU, GAAGACGGCGUGCACAAU, CCAAUCAGAUAGACUUCAU, M-006318-01) Qiagen: siMESH1-2 (target sequence CTGAAGGTCTCCTGCTAACTA, SI04167002). For transient knockdown, 60 pmole of siRNA and 9 μL of Lipofectamine RNAiMAX (ThermoFisher Scientific, #13778150) were reverse transfected together with 300 μL of opti-MEM (Gibco, #11058-021) to 2 × 10^5^ cells for 48–72 h. Empty vector for shRNA (empty) was purchased from Addgene (pLKO.1 puro, #8453). *MESH1* targeted shRNAs were purchased from Sigma: shMESH1-1 (target sequence TGAGGTGGAGCTACACTTTGG, TRCN0000243216), shMESH1-2 (target sequence TGGTGGAGGAGGTAACAGATG, and TRCN0000243217), shMESH1-3 (target sequence TCCATCCTTCCCAGATATTAG and TRCN0000243218). *AHR* targeted dox-inducible shRNAs were purchased from Horizon Discovery: shAHR-1 (ID: V3SH11252-227709444), shAHR-2 (ID: V3SH11252-230006145). For lentivirus package, 2 × 10^6^ HEK-293T cells were transfected with 4 μg of psPAX2 (Addgene, #12260), 0.4 μg of pMD2.G (Addgene, #12259), 4 μg of plasmid (empty or shRNAs), 24 μL of TransIT-LT1 (Mirus, MIR2305), and 800 μL of opti-MEM together with culture media for 48 h until the supernatant collection (virus soup). For stable shRNA overexpression, 10^5^ cells were infected with 500 μL of the virus soup together with 8 μg/mL polybrene for 24 h followed by the puromycin (1 μg/mL) selection continuously, and single-cell clones were generated after the selection until the validation of its overexpression on the western blot. For inducible shRNA knockdown, 2 μg/mL doxycycline was added to the culture media for at least 48 h before cell lysate collection or further dissipation in the tumor sphere formation assay (doxycycline was continuously added).

### TAZS89A and MESH1 overexpression

Lentiviral or retroviral plasmids were purchased from Addgene: empty (pBABE-puro, #1764); TAZS89A (pLenti-EF-FH-TAZS89A-ires-blast, #52084) [47]. MESH1-WT and MESH1-mutant (MESH1 E65A) was generated using the lentiviral backbone plasmid pLX302 (Addgene #25896).

### Cell culture

RCC4, HEK-293T, H1975, BT20, BT474, 786 O, SW-1353, MCF-7, and HT10801 were purchased from the Duke Cell Culture Facility and tested negative for mycoplasma. All cells except for the tumor sphere formation assay were cultured in the normal media: DMEM (ThermoFisher Scientific, 11995-DMEM,) 10% heat-inactivated fetal bovine serum (Hyclone #SH30070.03HI) and 1% pen-strep (ThermoFisher Scientific, #15140122) in a humidified incubator, at 37 °C with 5% CO_2_. For the tumor sphere formation, cells were cultured in the CCSC media: DMEM:F12 with l-glutamine (Invitrogen, #11330-032), 1% pen-strep (ThermoFisher Scientific, #15140122), 0.2x B27 supplement (Invitrogen, #17504044), 4 μg/mL heparin (Sigma, #H3149-50ku), 1x nonessential amino acid (Hyclone, #SH3023801), 1x sodium pyruvate (ThermoFisher Scientific, #11360-070), 40 ng/mL human EGF (R&D systems, #236-EG), 20 ng/mL bFGF (Invitrogen, #PHG0024), and 1 mL/100 mL N2 supplement (Invitrogen, #17502048).

### Western blot

Cell lysates were collected in the RIPA buffer (Sigma, R0278) with protease inhibitors (Roche, 11836170001) and protein concentrations were measured with BCA assay. For Western blots, 15–30 μg of protein were loaded on 8–15% SDS-PAGE gels, semi-dry transferred to the PDVF membrane, blocked with 5% milk in TBST, and then blotted with antibodies at 4 °C overnight. Antibodies were purchased from the anti-MESH1 antibody (Proteintech, 21091-1-AP); anti-β-tubulin antibody (Cell Signaling Technology, #2128); anti-YAP/TAZ antibody (Cell Signaling Technology, #8418); Anti-GAPDH antibody (Santa Cruz, sc-25778); anti-H3K27Ac antibody (Cell Signaling Technology, #8173); anti-mouse-IgG HRP (Cell Signaling Technology, #7076); and anti-rabbit-IgG HRP (Cell Signaling Technology, #7074). All primary antibodies were diluted 1:1000 in 5% BSA and secondary antibodies (anti-mouse-IgG HRP and anti-rabbit-IgG HRP) were diluted 1:2000 in 5% milk. Blots were developed with SuperSignal West Pico PLUS Chemiluminescent Substrate (ThermoFisher, #34577) and exposed in the ChemiDoc imaging system (Biorad). Each western blot image was repeated for three biologically independent times with similar trends.

### Quantitative real-time PCR

RNA was extracted with the RNeasy mini kit (Qiagen, #74104), reverse transcribed by the SuperScript II (ThermoFisher Scientific, #18064014) to generate cDNAs for qRT-PCR using primers and the Power SYBRGreen Mix (ThermoFisher Scientific, #4367659). Primers were designed across exon-exon junctions and the specificity of PCR products was checked by electrophoresis. *n* = 3 biologically independent replicates.

### Cell number, viability, and cell death measurement

For cell number count, cells were washed with cold PBS, trypsinized, treated with Trypan Blue (ThermoFisher Scientific, #15250061), and counted on the hemocytometer. *n* = 3 biologically independent replicates. For viability measurement, cells were treated with CellTiter-Glo^®^ assay reagents (Promega, #G7570) (*n* = 3 biologically independent replicates) or Crystal Violet reagents (*n* = 3 biologically independent replicates), dissolved by 10% acetic acid and quantified by the absorbance at 570 nm. Cell death was measured by the CellTox Green Cytotoxicity assay reagents (Promega, #G8741) (*n* = 3 biologically independent replicates) at 485/520 nm at 0, 16, 19, 24, 40, 48, 73, and 96 h post reagent addition by the FLUOstar Optima (BMG lab tech).

### Microarray, analysis, and GSEA analysis

Total RNAs were collected with RNeasy Mini Kit (Qiagen, #74104) and assessed with the Agilent BioAnalyzer. cDNAs were generated from 200 ng RNA using the Ambion MessageAmp Premier RNA Amplification (Life Technologies, Grand Island NY, USA) and were interrogated with Affymetrix U133A GeneChip. Gene expression data were deposited into NCBI GEO (GSE135358, GSE135346, and GSE147062). The microarray data were normalized by the RMA (Robust Multi-Array) algorithm. and zero transformed to the negative control (siNT), where we compared transcript levels for each gene in siMESH1 groups to the siNT group (*n* = 3 biologically independent replicates in each siRNA group) as previously performed [7, 9, 48]. Data were then filtered with Cluster 3.0 until ~1000 probesets were left and clustered by the gene. Heat maps were generated with TreeView with the indicated intensity. For GSEA analysis, siMESH1 vs. siNT microarray data were compared with the TAZ and YAP-induced genesets in the Supplementary table S1 in Zhang H, et al. [29]. (gene fold change ≤12-fold were cut off) using the Gene Set Enrichment Analysis (GSEA) performed at Broad Public Server with a default setting of 1000 permutations.

### BrdU incorporation assay

H1975 cells transduced with indicated inducible shRNAs were seeded in six-well plates and treated with doxycycline for 48 h, and then were transferred to four-well chamber slides (Sigma, #C6932-1PAK) 1 day prior to labeling. For labeling, cells were treated with 10 μM BrdU (ThermoFisher Scientific, #B23151) for 2 h at 37 °C, washed with warm PBS for 2 min three times, and fixed in 3.7% formaldehyde in PBS for 15 min at RT, followed by the incubation of permeabilization buffer (0.1% Triton X-100 in PBS) for 20 min. Permeabilized cells were buffer changed to 1 N HCl for 10 min on ice, 2 N HCl for 10 min at RT, phosphate/citric acid buffer (pH 7.4) for 10 min at RT, and washed with permeabilization buffer for 2 min three times. Processed cells were blocked with antibody staining solution (0.1% Triton X-100 + 5% normal goat serum in PBS) for 1 h at room temperature on a rocker and blotted with 1:100 anti-BrdU primary antibody (Abcam, #ab115874) overnight at 4 °C, followed by 1:500 anti-mouse Alexa-Flour 594 antibody (Abcam, #ab150116) for 1 h at RT, washed three times before being mounted with SlowFade Gold Antifade Mountant with DAPI (Invitrogen, #S36938), covered with the coverslips and imaged under a fluorescent microscope. *n* = 2 biologically independent replicates.

### PI (Propidium Iodide) stain and flow cytometry

For cell cycle analysis, 2 × 10^5^ H1975 cells were reverse transfected with the indicated siRNAs for 72 h, harvested using 0.05% trypsin with the media, fixed in 3 mL of ice-cold 70% ethanol, and gently vortexed until suspended. Fixed cells were centrifuged for 5 min at 1000 rpm at room temperature and washed twice with 3 mL of PBS at room temperature. For PI stain, cells were resuspended in 0.5–1 mL of PBS with 25 μg/mL PI (Sigma, #P4864) and 10 ug/mL RNAse A for 30 min at room temperature, light protected. At least 10^4^ processed cells were measured on a Canto II Flow cytometer at last. Data were analyzed using the FlowJo V10 software and the gating strategy was shown in Supplementary Fig. 4c. *n* = 3 biologically independent replicates.

### dNTP measurement

H1975 and RCC4 cells were transfected with the indicated siRNAs for 3 days, washed with ice-cold PBS twice, lysed by ice-cold 65% methanol, scrapped off, and extra 100 μL of 65% methanol were added to the plate to recover all the material. Lysates were vigorously vortexed for 2 min in 4 °C, incubated at 95 °C for 3 min, chilled on ice for 1 min, and centrifuge in 4 °C, 14,000 RPM for 3 min. The supernatant was dried by speed vacuum and analyzed in Kim’s lab at Emory University for dNTP quantification using HIV reverse transcriptase-based assay [49]. *n* = 3 biologically independent replicates.

### Animal study and xenograft

H1975 was transduced with empty vector (Tet-pLKO-puro, Addgene #21915, a gift from Dmitri Wiederschain) or shRNAs targeting MESH1 (shMESH1-1 and shMESH1-2) or AHR (shAHR-1 and shAHR-2). A single-cell colony for each shRNA was selected. About 1 × 10^6^ cells were injected subcutaneously in 0.1 ml of a 1:1 media to Matrigel (Corning, #354234) solution into the lower right flank of female immunodeficient mice (C.B-17 scid mice from Taconic lab) (empty vector: *n* = 7 biologically independent replicates, shMESH1-1: *n* = 9 biologically independent replicates, shMESH1-2: *n* = 8 biologically independent replicates, shMESH1-2 + TAZS89A OE: *n* = 9 biologically independent replicates, shAHR-1: *n* = 9 biologically independent replicates, shAHR-2: *n* = 9 biologically independent replicates). Once tumors were palpable (~1 week), all mice were switched to a doxycycline diet (Harlan, TD.110720, 2–3 mg doxycycline/day) for induction of shRNA. Tumor volumes were measured every other day using digital calipers until tumors reached 1.5–2 cm^3^, at which time mice were euthanized by carbon dioxide chamber, and tumors were harvested for weight measurement. Mice could also be euthanized if they became moribund or met other IACUC defined criteria suggesting pain or distress (e.g., weight loss >15%, ruffled fur, etc.) in the approved protocol by Duke IACUC (Registry Number A119-06-21.) During the course of xenograft injections and measurement, mice were handled randomly and blindly (only the ear numbers were given during the data collection process).

### Tumor sphere formation assay

About 0.7 × 10^5^ indicated H1975 cells were seeded in each well of the six-well plate in normal media, and 24 h later were treated with or without 2 μg/mL Doxycyclin for 48 h. A total of 7000 cells were then transferred to the 24-well low-attachment plate (Corning, #CLS3473) in CCSC media in replicates (*n* = 3) and were cultured for 10 days before counting. Control or doxycycline-containing CCSC media were added every 4 days to maintain knockdown. Tumor spheres were counted under the microscope by diameter size: II: 100–200 μm; III: 200–300 μm; IV: 300–400 μm; V: >400 μm. Three technical replicates were averaged to represent each biological replicate. *n* = 3 biologically independent replicates.

### Limited dilution assay

About 0.7 × 10^5^ indicated H1975 cells were seeded in a six-well plate in normal media and treated with or without 2 μg/mL Doxycycline for 48 h. Cells were then serial diluted to the 96-well round-bottom low-attachment plate (Corning, #7007) with 1000, 500, 100, 50 cells/well in CCSC media with technical replicates of 48 for each cell density. Spheres were cultured in CCSC media for 8 days before counting. Doxycycline-containing or deficient (no doxy control) CCSC media were added to each well every 4 days to maintain the knockdown level. After 8 days, the number of wells that successfully formed tumor sphere out of the 48 replicates for each cell density in each group was counted and data were plugged into the Extreme Limiting Dilution Analysis (ELDA) algorithm [50] for stem cell frequency, *p* value, and confidence interval calculations.

### ChIP-qPCR

Five million H1975 cells were seeded in 15 cm dishes and transfected with the siRNAs (siNT and siMESH1s) for 72 h before cross-link and collection of the cell lysates. Myers Lab ChIP-seq Protocol v011014-Adherent cells was then followed (https://www.encodeproject.org/documents/6ecd8240-a351-479b-9de6-f09ca3702ac3/@@download/attachment/ChIP-seq_Protocol_v011014.pdf). Sonication condition was optimized to the High mode, 30 s on/30 s off at 4 °C for 45 min using the Bioruptor Twin (Diagenode) sonicator. Dynabeads Protein G (Invitrogen, #10003D) and anti-H3K27Ac (Cell Signaling Technology, #8173), anti-TAZ (Cell Signaling Technology, #70148), anti-AHR (Santa Cruz Biotechnology, #sc-133088 X) or rabbit IgG (negative control: Santa Cruz Technology, sc-66931), mouse IgG (Santa Cruz Technology, sc-2025) were used for pull-down in this study as instructed. Pulled down DNA was then mixed with primers targeting the TAZ promoter, or YAP promoter, or TAZ-proximal heterochromatin region together with the Power SYBRGreen Mix (ThermoFisher Scientific, #4367659) to undergo the qPCR reaction as described above. Primers were designed following the guidelines on the Michigan University Nutritional Sciences website (http://bridgeslab.sph.umich.edu/protocols/index.php/RT-PCR_primer_design_for_ChIP). *n* = 3 biologically independent replicates.

### Co-immunoprecipitation

H1975 cells were transfected with the indicated vectors for 48 h. Cells were washed with PBS and resuspended in lysis buffer (50 mM Tris-HCl, 150 mM NaCl, 1% NP-40 and 5 mM EDTA, protease inhibitor cocktail, phosphatase inhibitor cocktail) at 4 °C with shaking for 30 min. Cell debris was pelleted by centrifugation at 16,000 × *g* for 15 min at 4 °C. Immunoprecipitation was performed using anti-FLAG (Anti-Flag M2 Affinity Gel, Sigma-Aldrich, #A2220) agarose beads at 4 °C overnight, washed three times and eluted using SDS loading buffer at 95 °C for 15 min, followed by the western blot using anti-HA (CST, #3724) and anti-FLAG (CST, #14793). *n* = 2 biologically independent replicates.

### MESH1 clinical relevance analysis

The expression of MESH1/HDDC3 as the probeset 227008_at (Affymetrix) or RNA-Seq were analyzed using prognostic database KM plotter, OncoLnc [51], and PROGgene V2.

### Statistical analysis and data collection

*n* numbers of biologically independent replicates were included in each figure legend and illustrated by the individual data points in each bar graph (mean + s.d.). The sample size was chosen based on our previous experience and literature reports for each experiment. *P* values were calculated by the indicated statistical analysis methods described in each figure legend and were justified as appropriate. Data collection was assigned blindly to different researchers.

## Supplementary information

Supplemental Figures

Reproducibility checklist

Video 1a

Video 1b

Video 1c

Supplemental Material-original western blots

## Data Availability

The data that support the findings of this study are available from the corresponding authors upon request. The microarray data have been deposited into NCBI GEO with accession numbers: GSE135358, GSE135346, and GSE147062.

## References

[1] Chi JT, Wang Z, Nuyten DS, Rodriguez EH, Schaner ME, Salim A (2006). Gene expression programs in response to hypoxia: cell type specificity and prognostic significance in human cancers. PLoS Med.

[2] Keenan MM, Liu B, Tang X, Wu J, Cyr D, Stevens RD (2015). ACLY and ACC1 regulate hypoxia-induced apoptosis by modulating ETV4 via alpha-ketoglutarate. PLoS Genet.

[3] Chen JL, Lucas JE, Schroeder T, Mori S, Wu J, Nevins J (2008). The genomic analysis of lactic acidosis and acidosis response in human cancers. PLoS Genet.

[4] Chen JL, Merl D, Peterson CW, Wu J, Liu PY, Yin H (2010). Lactic acidosis triggers starvation response with paradoxical induction of TXNIP through MondoA. PLoS Genet.

[5] Lamonte G, Tang X, Chen JL, Wu J, Ding CK, Keenan MM (2013). Acidosis induces reprogramming of cellular metabolism to mitigate oxidative stress. Cancer Metab.

[6] Gatza ML, Kung HN, Blackwell KL, Dewhirst MW, Marks JR, Chi JT (2011). Analysis of tumor environmental response and oncogenic pathway activation identifies distinct basal and luminal features in HER2-related breast tumor subtypes. Breast Cancer Res.

[7] Tang X, Ding CK, Wu J, Sjol J, Wardell S, Spasojevic I (2017). Cystine addiction of triple-negative breast cancer associated with EMT augmented death signaling. Oncogene.

[8] Tang X, Keenan MM, Wu J, Lin CA, Dubois L, Thompson JW (2015). Comprehensive profiling of amino acid response uncovers unique methionine-deprived response dependent on intact creatine biosynthesis. PLoS Genet.

[9] Tang X, Wu J, Ding CK, Lu M, Keenan MM, Lin CC (2016). Cystine deprivation triggers programmed necrosis in VHL-deficient renal cell carcinomas. Cancer Res.

[10] Potrykus K, Cashel M (2008). (p)ppGpp: still magical?. Annu Rev Microbiol.

[11] Kriel A, Bittner AN, Kim SH, Liu K, Tehranchi AK, Zou WY (2012). Direct regulation of GTP homeostasis by (p)ppGpp: a critical component of viability and stress resistance. Mol Cell.

[12] Wang B, Dai P, Ding D, Del Rosario A, Grant RA, Pentelute BL (2019). Affinity-based capture and identification of protein effectors of the growth regulator ppGpp. Nat Chem Biol.

[13] Sun D, Lee G, Lee JH, Kim HY, Rhee HW, Park SY (2010). A metazoan ortholog of SpoT hydrolyzes ppGpp and functions in starvation responses. Nat Struct Mol Biol.

[14] Ding C-KC, Rose J, Sun T, Wu J, Chen P-H, Lin C-C (2020). MESH1 is a cytosolic NADPH phosphatase that regulates ferroptosis. Nature. Metabolism.

[15] Khan S, Greco D, Michailidou K, Milne RL, Muranen TA, Heikkinen T (2014). MicroRNA related polymorphisms and breast cancer risk. PLoS One.

[16] Bild AH, Yao G, Chang JT, Wang Q, Potti A, Chasse D (2006). Oncogenic pathway signatures in human cancers as a guide to targeted therapies. Nature.

[17] Tripathi MK, Deane NG, Zhu J, An H, Mima S, Wang X (2014). Nuclear factor of activated T-cell activity is associated with metastatic capacity in colon cancer. Cancer Res.

[18] Lee Y, Liu J, Patel S, Cloughesy T, Lai A, Farooqi H (2010). Genomic landscape of meningiomas. Brain Pathol.

[19] Leich E, Salaverria I, Bea S, Zettl A, Wright G, Moreno V (2009). Follicular lymphomas with and without translocation t(14;18) differ in gene expression profiles and genetic alterations. Blood..

[20] Durfee T, Hansen AM, Zhi H, Blattner FR, Jin DJ (2008). Transcription profiling of the stringent response in Escherichia coli. J Bacteriol.

[21] Reichard P (1988). Interactions between deoxyribonucleotide and DNA synthesis. Annu Rev Biochem.

[22] Nordlund P, Reichard P (2006). Ribonucleotide reductases. Annu Rev Biochem.

[23] Cordenonsi M, Zanconato F, Azzolin L, Forcato M, Rosato A, Frasson C (2011). The Hippo transducer TAZ confers cancer stem cell-related traits on breast cancer cells. Cell..

[24] Zhao B, Lei QY, Guan KL (2008). The Hippo-YAP pathway: new connections between regulation of organ size and cancer. Curr Opin Cell Biol.

[25] Zanconato F, Cordenonsi M, Piccolo S (2016). YAP/TAZ at the roots of cancer. Cancer Cell.

[26] Yang W-H, Lin C-C, Wu J, Chao P-Y, Chen K, Chen P-H (2021). The Hippo pathway effector YAP promotes ferroptosis via the E3 ligase SKP2. Mol Cancer Res.

[27] Yang W-H, Ding C-KC, Sun T, Rupprecht G, Lin C-C, Hsu D (2019). pathway effector TAZ regulates ferroptosis in renal cell carcinoma. Cell Rep.

[28] Yang WH, Huang Z, Wu J, Ding C-KC, Murphy SK, Chi J-T (2019). A TAZ-ANGPTL4-NOX2 axis regulates ferroptotic cell death and chemoresistance in epithelial ovarian cancer. Mol Cancer Res.

[29] Zhang H, Liu CY, Zha ZY, Zhao B, Yao J, Zhao S (2009). TEAD transcription factors mediate the function of TAZ in cell growth and epithelial-mesenchymal transition. J Biol Chem.

[30] Lin CC, Ding CC, Sun T, Wu J, Chen KY, Zhou P (2021). The regulation of ferroptosis by MESH1 through the activation of the integrative stress response. Cell Death Dis.

[31] Lei Q-Y, Zhang H, Zhao B, Zha Z-Y, Bai F, Pei X-H (2008). TAZ promotes cell proliferation and epithelial-mesenchymal transition and is inhibited by the Hippo pathway. Mol Cell Biol.

[32] Zanconato F, Forcato M, Battilana G, Azzolin L, Quaranta E, Bodega B (2015). Genome-wide association between YAP/TAZ/TEAD and AP-1 at enhancers drives oncogenic growth. Nat Cell Biol.

[33] Fishilevich S, Nudel R, Rappaport N, Hadar R, Plaschkes I, Iny Stein T (2017). GeneHancer: genome-wide integration of enhancers and target genes in GeneCards. Database.

[34] Konermann S, Brigham MD, Trevino AE, Joung J, Abudayyeh OO, Barcena C (2015). Genome-scale transcriptional activation by an engineered CRISPR-Cas9 complex. Nature.

[35] Glozak MA, Seto E (2007). Histone deacetylases and cancer. Oncogene.

[36] Haberland M, Montgomery RL, Olson EN (2009). The many roles of histone deacetylases in development and physiology: implications for disease and therapy. Nat Rev Genet.

[37] Stark C, Breitkreutz BJ, Reguly T, Boucher L, Breitkreutz A, Tyers M (2006). BioGRID: a general repository for interaction datasets. Nucleic Acids Res.

[38] CLC Bio QIAGEN. White paper on the transcription factor ChIP-seq. 2015. https://resources.qiagenbioinformatics.com/white-papers/Transcription_factor_ChIP-seq.pdf?_ga=2.219229657.1909906395.1620224188-1671251113.1620224188.

[39] Oshima M, Mimura J, Sekine H, Okawa H, Fujii-Kuriyama Y (2009). SUMO modification regulates the transcriptional repressor function of aryl hydrocarbon receptor repressor. J Biol Chem.

[40] Hong W, Guan KL (2012). The YAP and TAZ transcription co-activators: key downstream effectors of the mammalian Hippo pathway. Semin Cell Dev Biol.

[41] Sun T, Chi J-T (2020). Regulation of ferroptosis in cancer cells by YAP/TAZ and Hippo pathways: the therapeutic implications. Genes Dis.

[42] Dupont S, Morsut L, Aragona M, Enzo E, Giulitti S, Cordenonsi M (2011). Role of YAP/TAZ in mechanotransduction. Nature..

[43] Zhao B, Wei X, Li W, Udan RS, Yang Q, Kim J (2007). Inactivation of YAP oncoprotein by the Hippo pathway is involved in cell contact inhibition and tissue growth control. Genes Dev.

[44] Liu CY, Yu T, Huang Y, Cui L, Hong W (2017). ETS (E26 transformation-specific) up-regulation of the transcriptional co-activator TAZ promotes cell migration and metastasis in prostate cancer. J Biol Chem.

[45] Liu CY, Chan SW, Guo F, Toloczko A, Cui L, Hong W (2016). MRTF/SRF dependent transcriptional regulation of TAZ in breast cancer cells. Oncotarget..

[46] Hilman D, Gat U (2011). The evolutionary history of YAP and the hippo/YAP pathway. Mol Biol Evol.

[47] Yang Z, Nakagawa K, Sarkar A, Maruyama J, Iwasa H, Bao Y (2014). Screening with a novel cell-based assay for TAZ activators identifies a compound that enhances myogenesis in C2C12 cells and facilitates muscle repair in a muscle injury model. Mol Cell Biol.

[48] Lin CC, Mabe NW, Lin YT, Yang WH, Tang X, Hong L (2020). RIPK3 upregulation confers robust proliferation and collateral cystine-dependence on breast cancer recurrence. Cell Death Differ.

[49] Diamond TL, Roshal M, Jamburuthugoda VK, Reynolds HM, Merriam AR, Lee KY (2004). Macrophage tropism of HIV-1 depends on efficient cellular dNTP utilization by reverse transcriptase. J Biol Chem.

[50] Hu Y, Smyth GK (2009). ELDA: extreme limiting dilution analysis for comparing depleted and enriched populations in stem cell and other assays. J Immunol Methods.

[51] Anaya. (2016). OncoLnc: linking TCGA survival data to mRNAs, miRNAs, and lncRNAs. PeerJ Comput Sci..

