# Supplementary material for: MESH1 knockdown triggers proliferation arrest through TAZ repression
Source: Cell Death Dis. 2022 Mar 10;13(3):221. doi: 10.1038/s41419-022-04663-6 (PMC8913805; doi:10.1038/s41419-022-04663-6)

Supplemental Fig 1

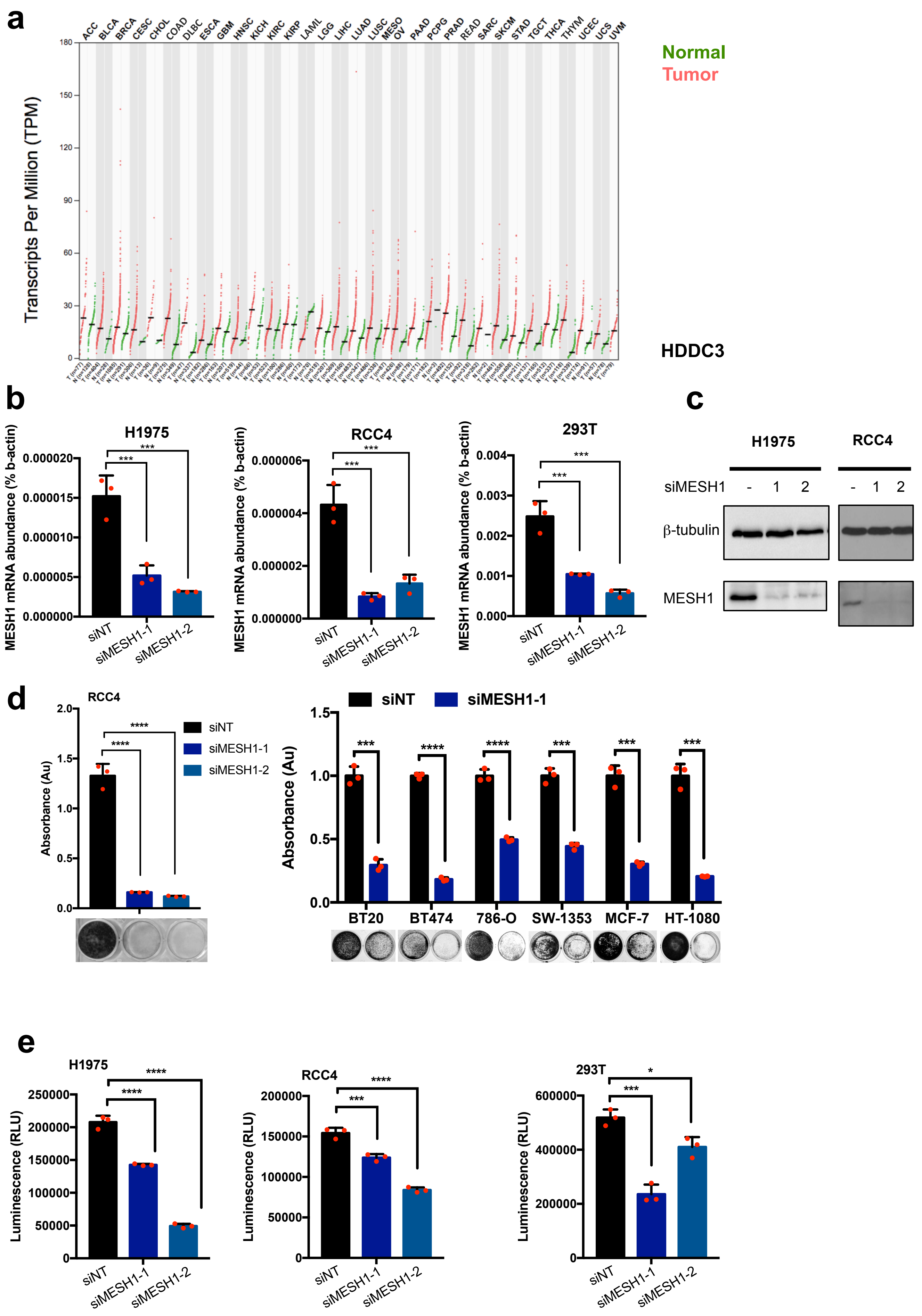

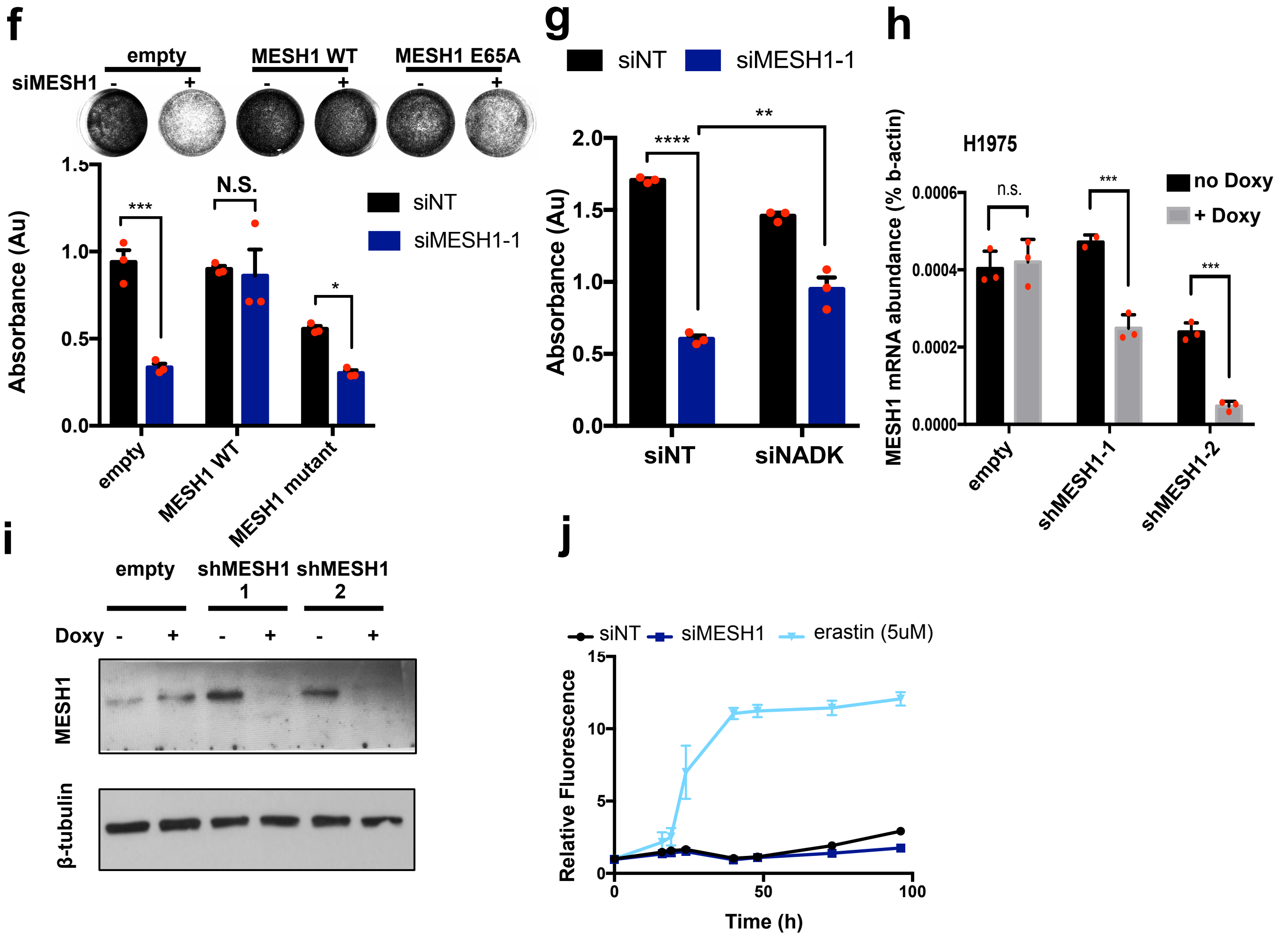

Supplemental Figure 1. *MESH1* knockdown led to cell proliferation arrests in multiple cancer cell lines. a) Expression (RNA-seq from TCGA) analysis of HDDC3 in a panel of tumor and normal patient samples suggested that tumor cells tend to express higher level of HDDC3 compared to normal cells. **b)** *MESH1* mRNA level and **c)** protein expression was inhibited upon siRNA introduction in H1975, RCC4 and 293T cells. (mean+s.d.). **d)** Representative image of the crystal violet staining (bottom) and quantification for replicates (up) of RCC4, BT20, BT474, 786-O, SW-1353, MCF-7, and HT-1080 cells showed consistent cell number reduction upon *MESH1* knockdown. Higher absorbance represents higher cell numbers. (mean+s.d.). **e)** Measurement of the cell viability by CellTiter-Glo® Assay revealed the inhibition of cell growth upon *MESH1* knockdown in H1975, RCC4, and 293T cells. Higher luminescence signals represent higher viability. (mean+s.d.). **f)** Representative image of the crystal violet staining (top) and quantification for replicates (bottom) of H1975 cells showed that wildtype (WT), but not mutant, MESH1 expression restored the cell number reduction upon *MESH1* knockdown, suggesting the specificity of this phenotype (mean+s.d.). **g)** Quantification for the crystal violet staining of H1975 cells showed a restoration of cell number by *NADK* knockdown, suggesting the importance of MESH1 enzymatic activity. (mean+s.d.). **h)** qRT-PCR validation of the *MESH1* mRNA depletion upon the doxycycline induction of shMESH1 in H1975 cells. (mean+s.d.). **i)** Representative images of the western blots validated that MESH1 protein level was reduced upon the doxycycline induction of shMESH1 in H1975 cells. **j)** Measurement of the DNA release and cell death by CellTox Green Assay revealed that siMESH1 had no effect on cell death in H1975 cells. Erastin (5uM) served as the positive control for the cell death signals. (mean±s.d.). For b); d); e), p values were calculated by the one-way ANOVA followed by the Tukey's post test. For f); g); h), p values were calculated by the two-way ANOVA followed by the Tukey's post test. * *P*~(0.01, 0.05); ** *P*~(0.001, 0.01); *** *P*~(0.0001, 0.001); **** *P*<0.0001; N.S. no significance.

Supplemental Fig 2

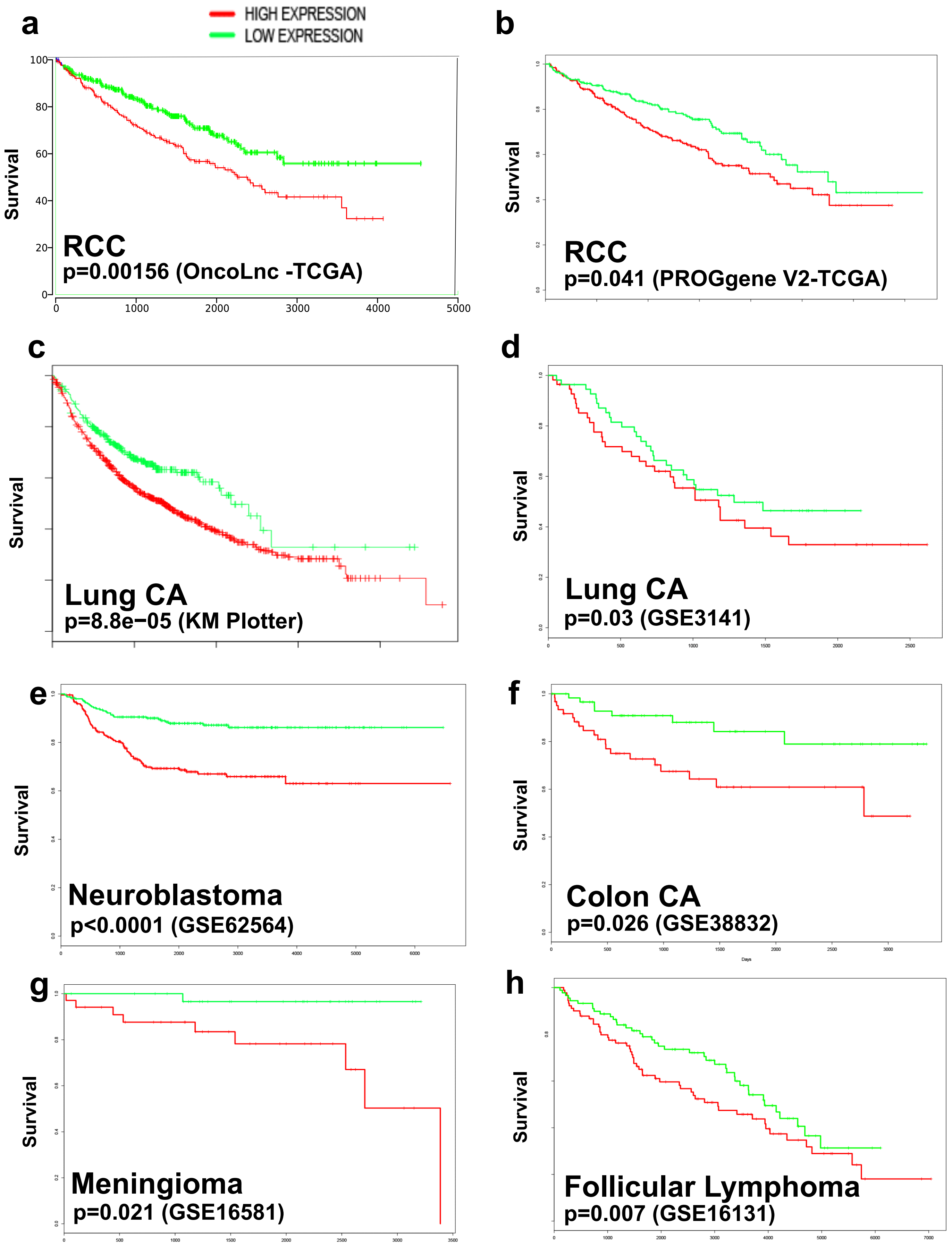

Supplemental Figure 2. The prognostic significance of MESH1 in human tumors. Patients' data with **a)** renal cell carcinoma (OncoLnc-TCGA), **b)** clear cell renal cell carcinoma (PROGgene V2-TCGA), **c)** lung cancer (KM Plotter), **d)** lung cancer (GSE3141), **e)** neuroblastoma (GSE62564), **f)** colon cancer (GSE38832), **g)** meningioma (GSE16581), **h)** follicular lymphoma (GSE16131) in the indicated datasets stratified by the expression of HDDC3 (encoding MESH1) were used to generate Kaplan-Meier survival curves with indicated statistical significance to link clinical outcomes with the HDDC3 expression levels. Higher levels of MESH1 are associated with poor survival of the patients in these tumor datasets.

Supplemental Fig 3

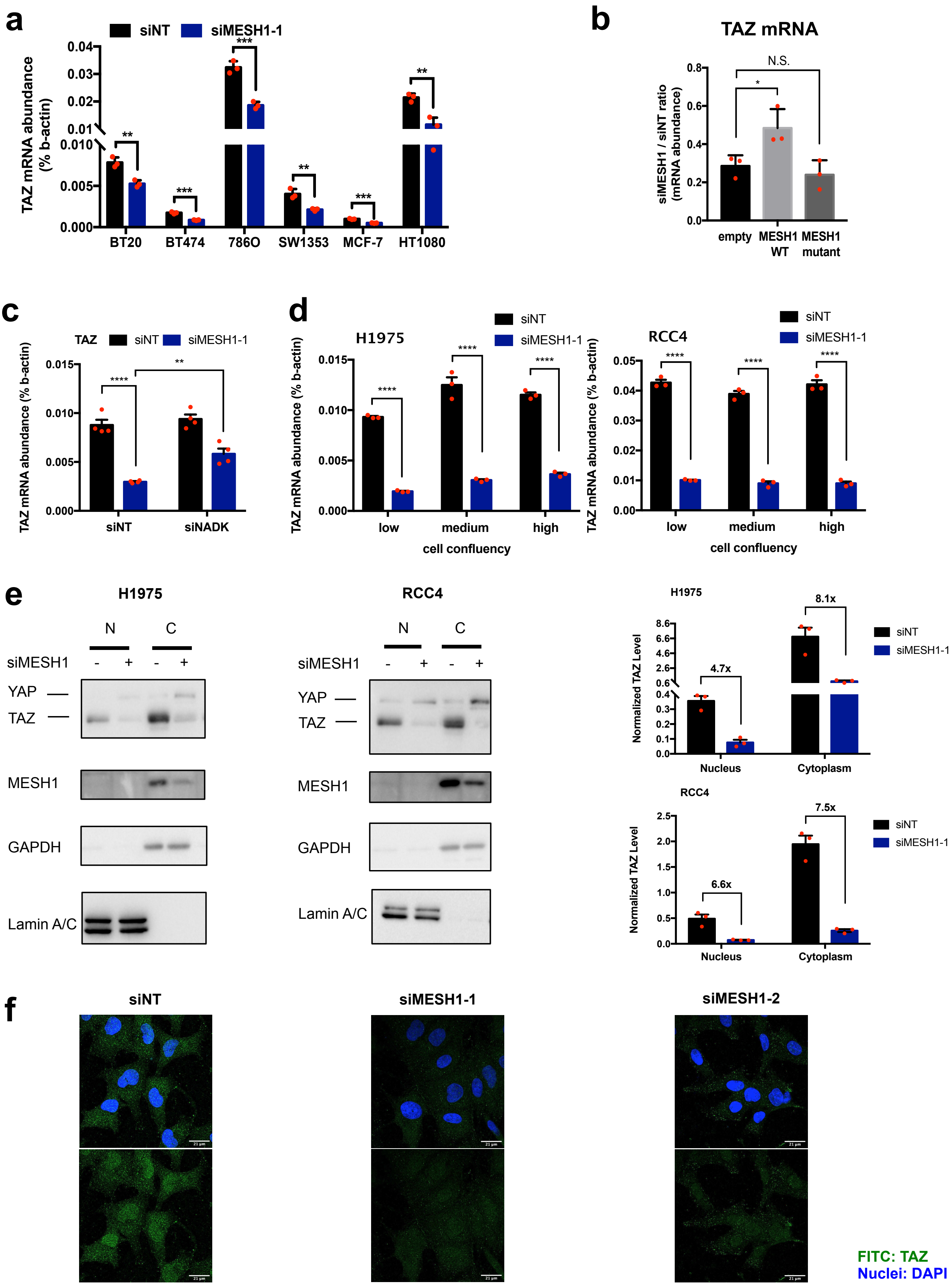

g

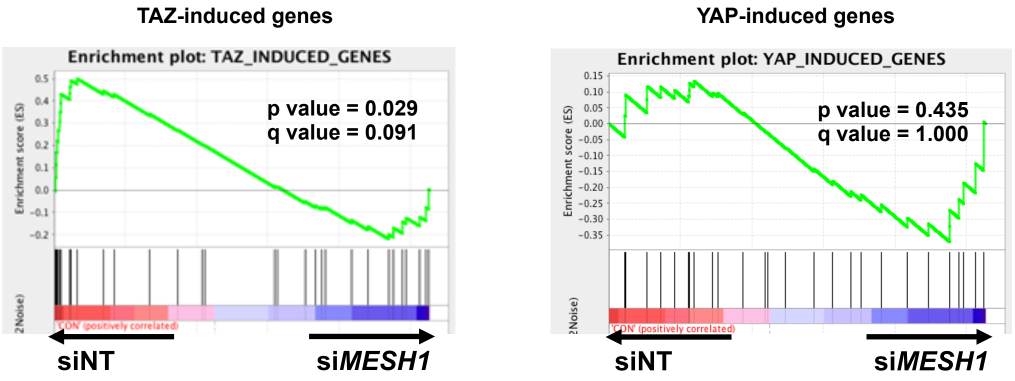
TAZ-induced genes
Enrichment plot: TAZ_INDUCED_GENES
Enrichment score (ES)
p value = 0.029
q value = 0.091
siNT
siMESH1
YAP-induced genes
Enrichment plot: YAP_INDUCED_GENES
Enrichment score (ES)
p value = 0.435
q value = 1.000
siNT
siMESH1

h

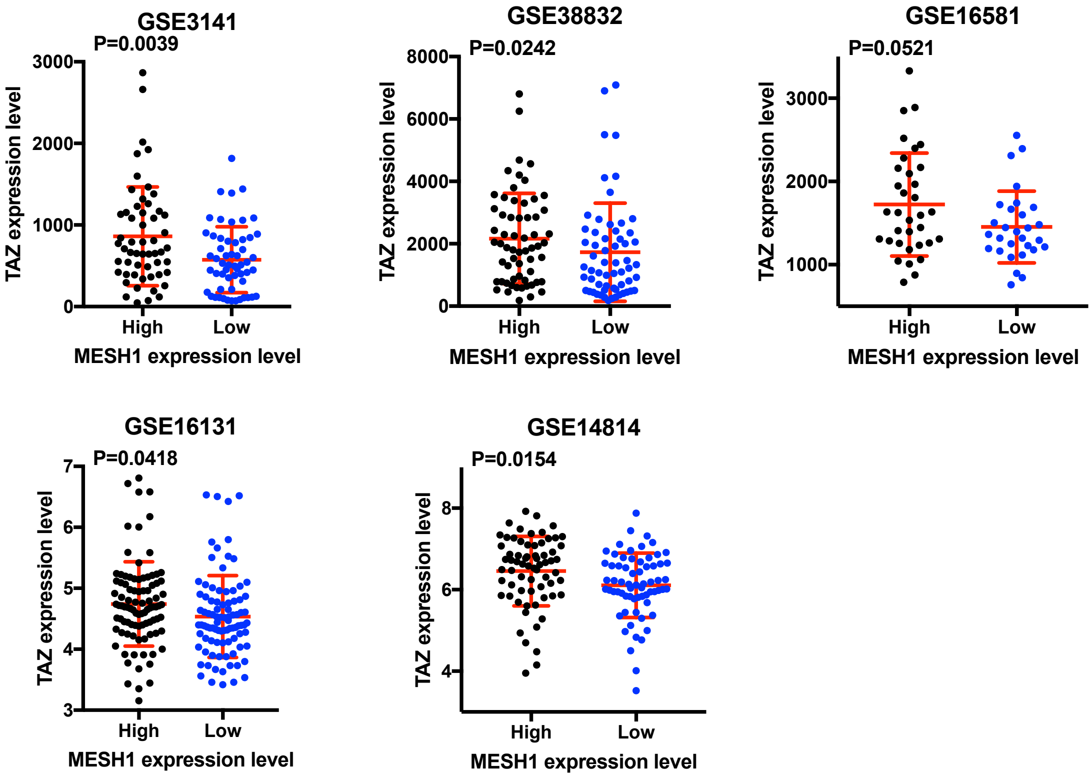
GSE3141
P=0.0039
TAZ expression level
High
Low
MESH1 expression level
GSE38832
P=0.0242
TAZ expression level
High
Low
MESH1 expression level
GSE16581
P=0.0521
TAZ expression level
High
Low
MESH1 expression level
GSE16131
P=0.0418
TAZ expression level
High
Low
MESH1 expression level
GSE14814
P=0.0154
TAZ expression level
High
Low
MESH1 expression level

Supplemental Figure 3. *MESH1* knockdown repressed *TAZ* transcription and its activity. **a)** *TAZ* mRNA expression tested by qRT-PCR was repressed upon *MESH1* knockdown in BT20, BT474, 786-O, SW-1353, MCF-7, and HT-1080 cells. (mean+s.d.). **b)** qRT-PCR revealed that the *TAZ* mRNA repression upon *MESH1* knockdown was mitigated by the wildtype *MESH1* restoration and **c)** *NADK* knockdown in H1975 cells. *MESH1* enzymatic mutant expression failed to rescue the *TAZ* repression. (mean+s.d.). **d)** qRT-PCR revealed that *MESH1* knockdown repressed *TAZ* mRNA level regardless of the cell density in H1975 and RCC4 cells. In H1975, ”low”, “medium”, and ”high” denote 10^5, 2x10^5, and 3x10^5 cells respectively; In RCC4, ”low”, “medium”, and ”high” denote 0.5x10^5, 10^5, and 1.5x10^5 cells respectively. (mean+s.d.). **e)** Representative images of western blots (left) and its quantification (right) revealed that *MESH1* knockdown repressed TAZ protein to similar degrees in cytosol and nucleus in H1975 and RCC4 cells. N represents nucleus; C represents cytosol. Nuclear TAZ levels were normalized to the lamin A/C levels; Cytosolic TAZ levels were normalized to the GAPDH levels. (mean+s.d.). **f)** Representative images of immunofluorescence staining revealed that *MESH1* knockdown repressed TAZ protein level both in the cytosol and nucleus in H1975 cells. Up: merged; Down: TAZ signal. Scale bars: 21μm. **g)** GSEA analysis of the inhibition of TAZ-, but not YAP-induced gene signatures in siMESH1 H1975 cells. The TAZ and YAP-induced gene signatures were defined in the method [19]. TAZ: p value = 0.029, q value = 0.091; YAP: p value = 0.435, q value = 1.000. **h)** Gene expression analyses suggested that patients with low MESH1 level displayed low TAZ expression in the indicated datasets. Patients’ gene expression data from the indicated datasets were stratified by MESH1 levels into “High” and “Low” expression groups 50/50 and their corresponding TAZ levels were shown on the y-axis (mean±s.d.). For b), p values were calculated by the one-way ANOVA followed by the Tukey’s post test. For a); c); d), p values were calculated by the two-way ANOVA followed by the Tukey’s post test. For h), p values were calculated by the student’s t-test. * *P*~(0.01, 0.05); ** *P*~(0.001, 0.01); **** *P*<0.0001; N.S. no significance.

Supplemental Figure 4

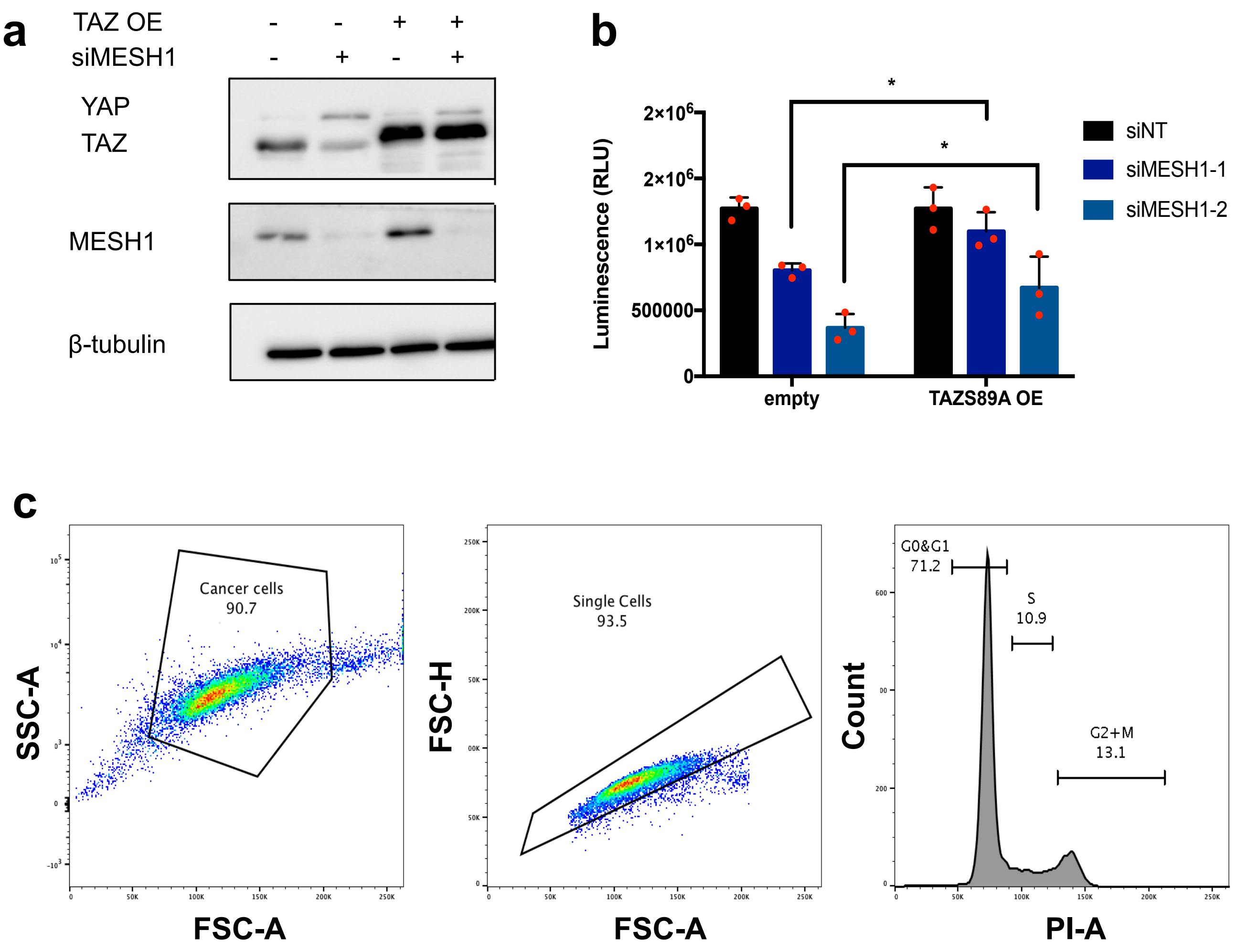

Supplemental Figure 4. *TAZ* restoration rescued proliferation arrest triggered by *MESH1* knockdown. **a)** Representative images of the western blots validated the successful over-expression of TAZS89A and *MESH1* knockdown in H1975 cells. **b)** Measurement of the cell viability by CellTiter Glo® Assay revealed that *TAZ* restoration significantly rescued the inhibition of cell growth by siMESH1 in H1975 cells. (mean+s.d., p values were calculated by the two-way ANOVA followed by the Tukey's post test. * *P*~(0.01, 0.05)). **c)** Representative images showed the gating strategy for cell cycle analysis using the FlowJo.

Supplemental Figure 5

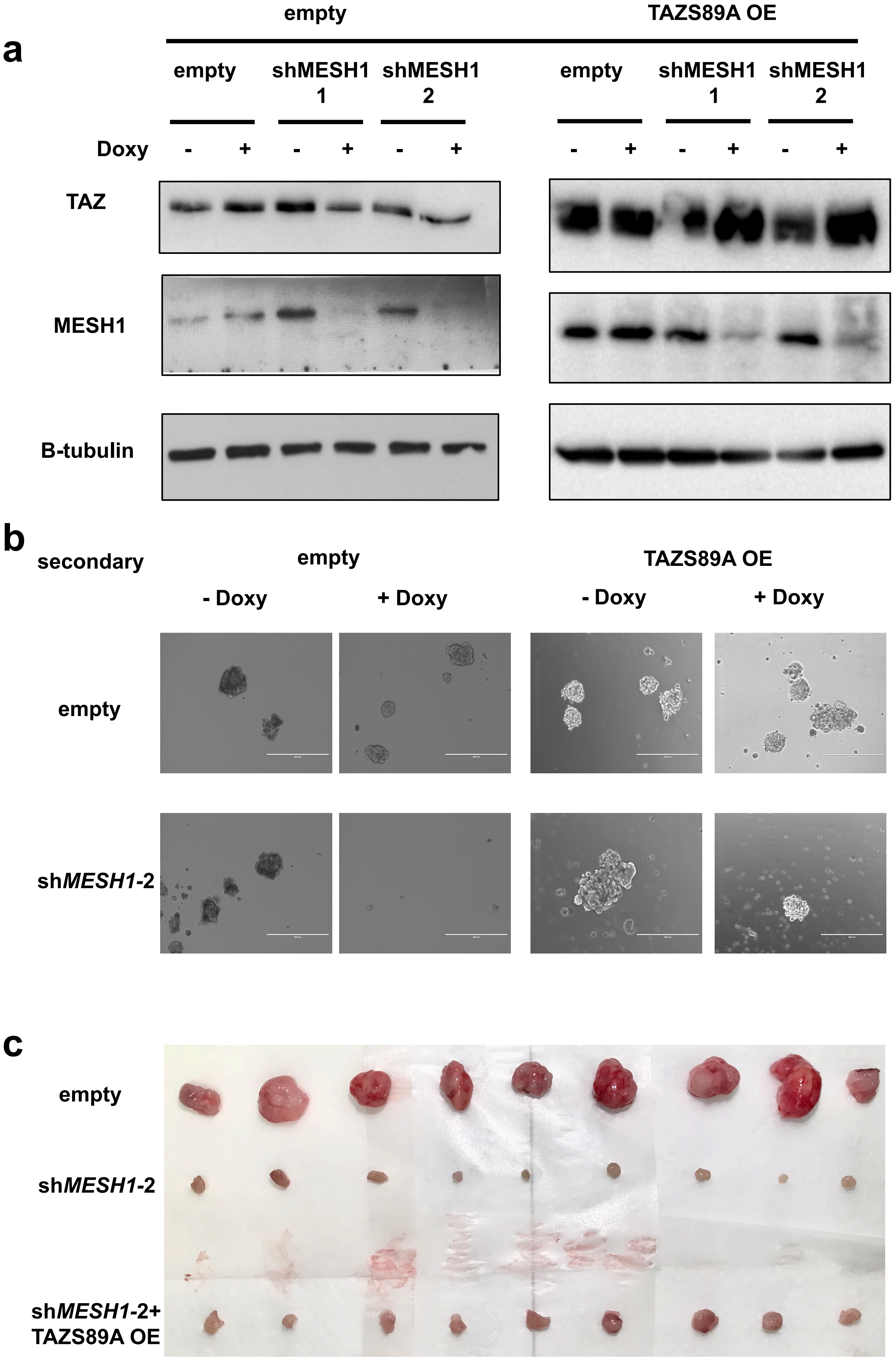

Supplemental Figure 5. *TAZ* restoration rescued the self-renewal capacity inhibited by *MESH1* knockdown in H1975 cells. **a)** Representative images of the western blots validated the repression of TAZ protein by *MESH1* knockdown and its restored expression upon the transfection of TAZS89A. **b)** Representative images of the secondary tumor sphere formation assay revealed that TAZS89A expression upon *MESH1* knockdown continuously increased tumor sphere numbers and sizes in H1975. **c)** Images of the harvested xenograft tumors of different treatment groups from Fig 3i.

Supplemental Figure 6

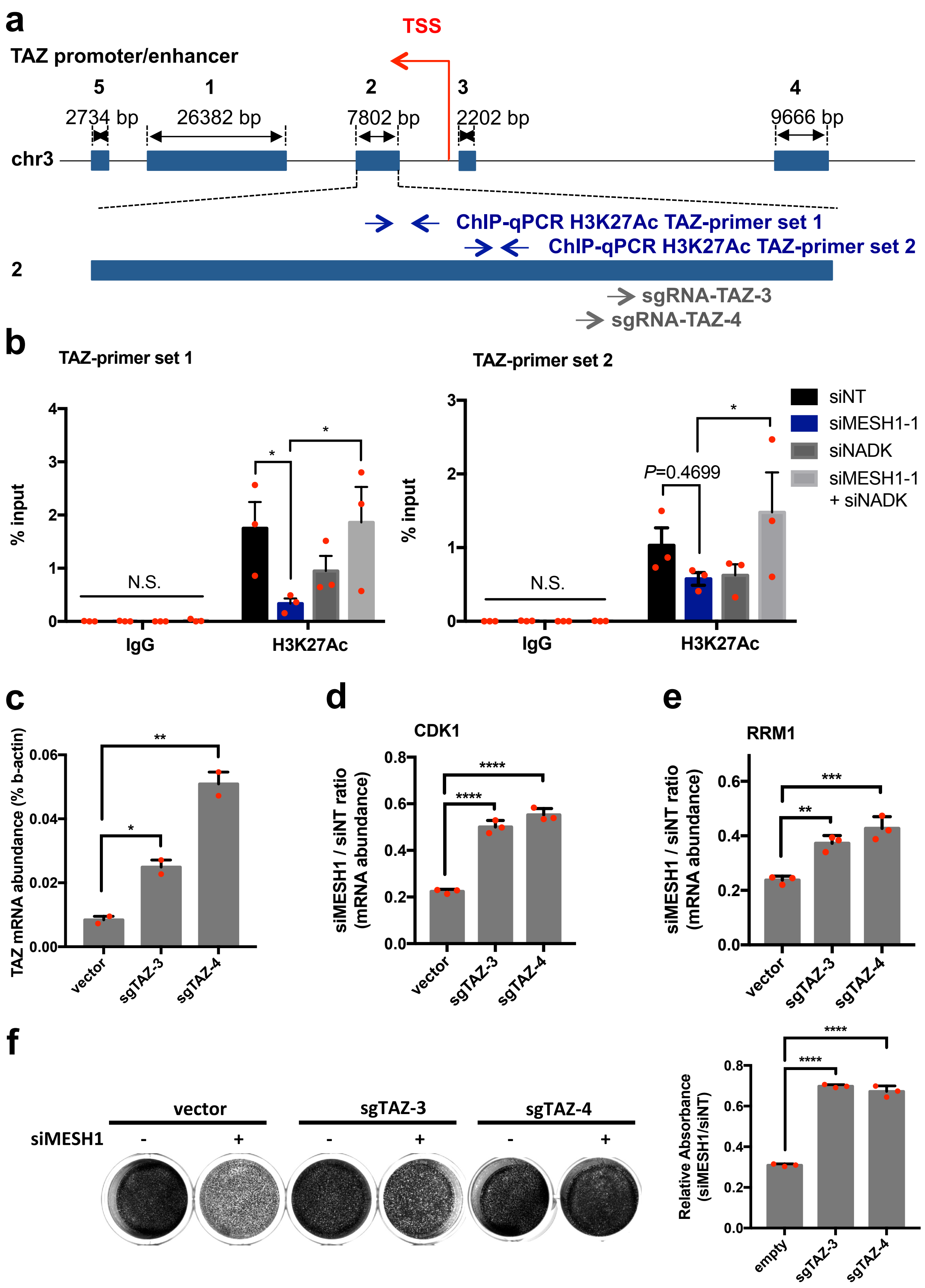

Supplemental Figure 6. CRISPRa activation of TAZ rescued cell growth inhibited by *MESH1* knockdown. **a)** Representative map of TAZ promoter/enhancer regions annotated by GeneHancer and the locations of primers for the H3K27Ac ChIP-qPCR and CRISPR-a experiments. **b)** ChIP-qPCR analysis of the abundance of the H3K27Ac mark at TAZ promoter region in cells transfected with the control (siNT), *MESH1* siRNA (siMESH1-1), *NADK* siRNA (siNADK), or both (siMESH1-1+siNADK). (mean+s.d.). **c)** qRT-PCR validation of the enhanced transcription of *TAZ* by the CRISPRa system. H1975 cells were first stably transfected with the VP64 and MS2-P65-HSF1 as the vector group and then transfected with the indicated sgRNAs targeting TAZ regulatory regions. (mean+s.d.). **d)** qRT-PCR revealed that *TAZ* activation by CRISPRa significantly rescued the *CDK1* repression caused by *MESH1* knockown. (mean+s.d.). **e)** qRT-PCR revealed that *TAZ* activation by CRISPRa significantly rescued the *RRM1* repression caused by *MESH1* knockdown. (mean+s.d.). **f)** Representative images of the crystal violet staining and quantification (right) revealed that *TAZ* activation by CRISPRa significantly rescued the cell number reduced by *MESH1* knockdown. (mean+s.d.). For b), p values were calculated by the two-way ANOVA followed by the Tukey's post test. For c); d); e); f), p values were calculated by the one-way ANOVA followed by the Tukey's post test. * *P*~(0.01, 0.05); ** *P*~(0.001, 0.01); **** *P*<0.0001; N.S. no significance.

Supplemental Figure 7

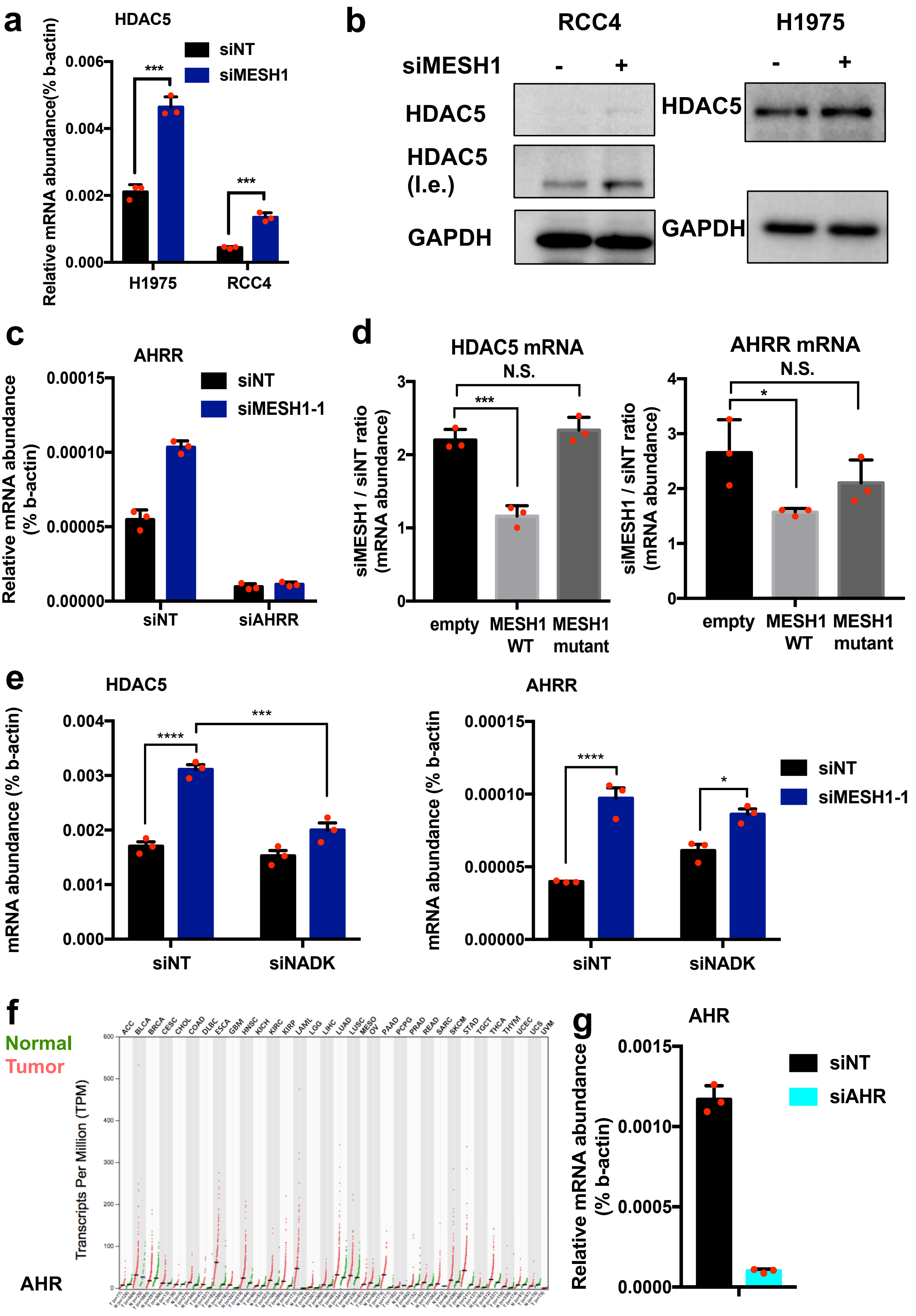

h

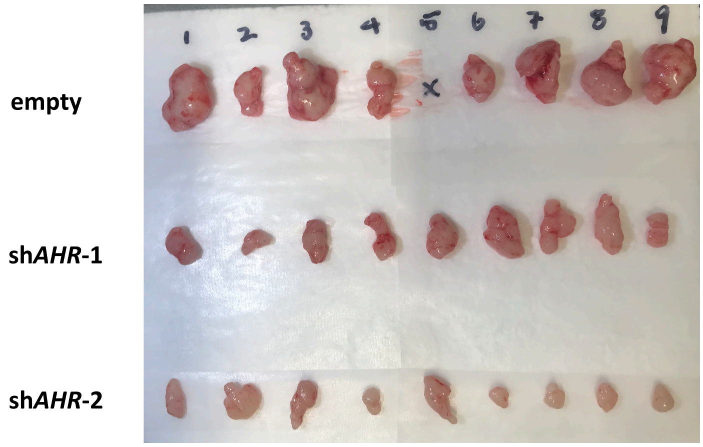

empty
shAHR-1
shAHR-2
1 2 3 4 5 6 7 8 9

i

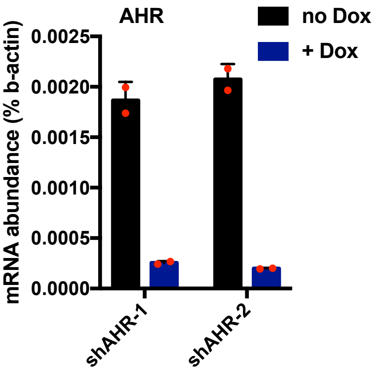

AHR
no Dox
+ Dox
mRNA abundance (% b-actin)
0.0025
0.0020
0.0015
0.0010
0.0005
0.0000
shAHR-1
shAHR-2

j

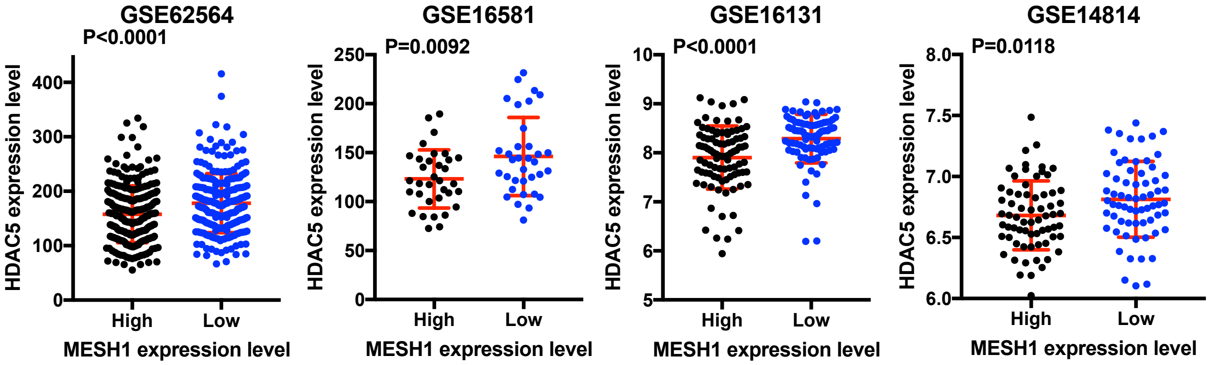

GSE62564
P<0.0001
GSE16581
P=0.0092
GSE16131
P<0.0001
GSE14814
P=0.0118
HDAC5 expression level
High
Low
MESH1 expression level

k

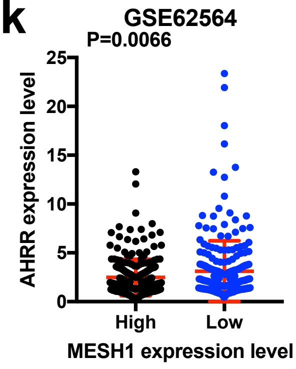

GSE62564
P=0.0066
AHRR expression level
High
Low
MESH1 expression level

Supplemental Figure 7. HDAC5 and AHRR contributed to the *TAZ* transcriptional repression upon *MESH1* knockdown. **a)** qRT-PCR validation of *HDAC5* up-regulation by *MESH1* knockdown in H1975 and RCC4 cells. (mean+s.d., p values were calculated by the student's t-test. *** *P*~(0.0001, 0.001)). **b)** Representative image of the western blot validated the up-regulation of HDAC5 protein by *MESH1* knockdown in both H1975 and RCC4 cells. **c)** qRT-PCR validation of *AHRR* up-regulation by *MESH1 knockdown* and the successful knockdown of *AHRR.* (mean+s.d.). **d)** qRT-PCR revealed that the *HDAC5* and *AHRR* mRNA up-regulation upon *MESH1* knockdown was restored by the wildtype *MESH1* restoration in H1975 cells. *MESH1* enzymatic mutant expression failed to restore the *HDAC5* and *AHRR* increase. (mean+s.d.). **e)** qRT-PCR revealed that the *HDAC5* (left) and *AHRR* (right) mRNA up-regulation upon *MESH1* knockdown was restored by *NADK* knockdown in H1975 cells. (mean+s.d.). **f)** Expression (RNA-seq from TCGA) analysis of AHR in a panel of tumor and normal patient samples suggested that tumor cells tend to express higher level of AHR compared to normal cells**. g)** qRT-PCR validation of the *AHR* knockdown by siAHR. (mean+s.d.). **h)** Image of the harvested xenograft tumors of different treatments from Fig 5j. Mouse #5 in the empty group died before the final end point and was eliminated from the analysis. **i)** qPCR validation of *AHR* inducible knockdown in H1975 injected in the xenografts in Fig 5j. **j) k)** Gene expression analyses indicated that tumors with low MESH1 level displayed high HDAC5 **(j)** and AHRR **(k)** expression in the indicated tumor datasets. Patients' gene expression data from the indicated datasets were stratified by MESH1 levels into "High" and "Low" expression groups 50/50 and their corresponding HDAC5 and AHRR levels were shown on the y-axis (mean±s.d.). For d), p values were calculated by the one-way ANOVA followed by the Tukey's post test. For e), p values were calculated by the two-way ANOVA followed by the Tukey's post test. For j), k), p values were calculated by the student's t-test. * *P*~(0.01, 0.05); *** *P*~(0.0001, 0.001); **** *P*<0.0001; N.S. no significance.

Supplemental Figure 8 Model

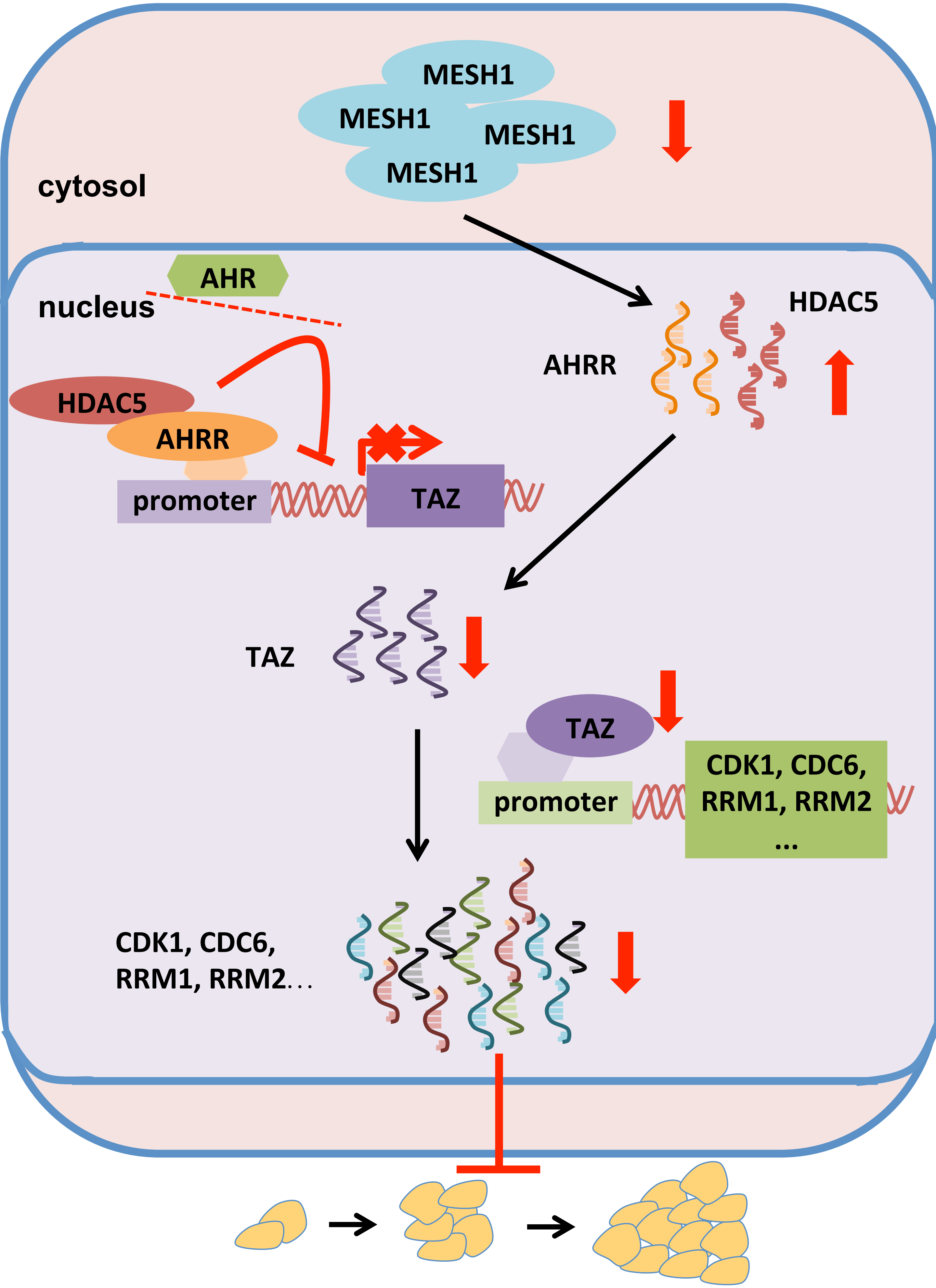

Supplement: Supplementary file 1 — Supplemental Figures [file 41419_2022_4663_MOESM1_ESM.pdf]
