# Supplementary material for: MESH1 knockdown triggers proliferation arrest through TAZ repression
Source: Cell Death Dis. 2022 Mar 10;13(3):221. doi: 10.1038/s41419-022-04663-6 (PMC8913805; doi:10.1038/s41419-022-04663-6)

Figure 2

f

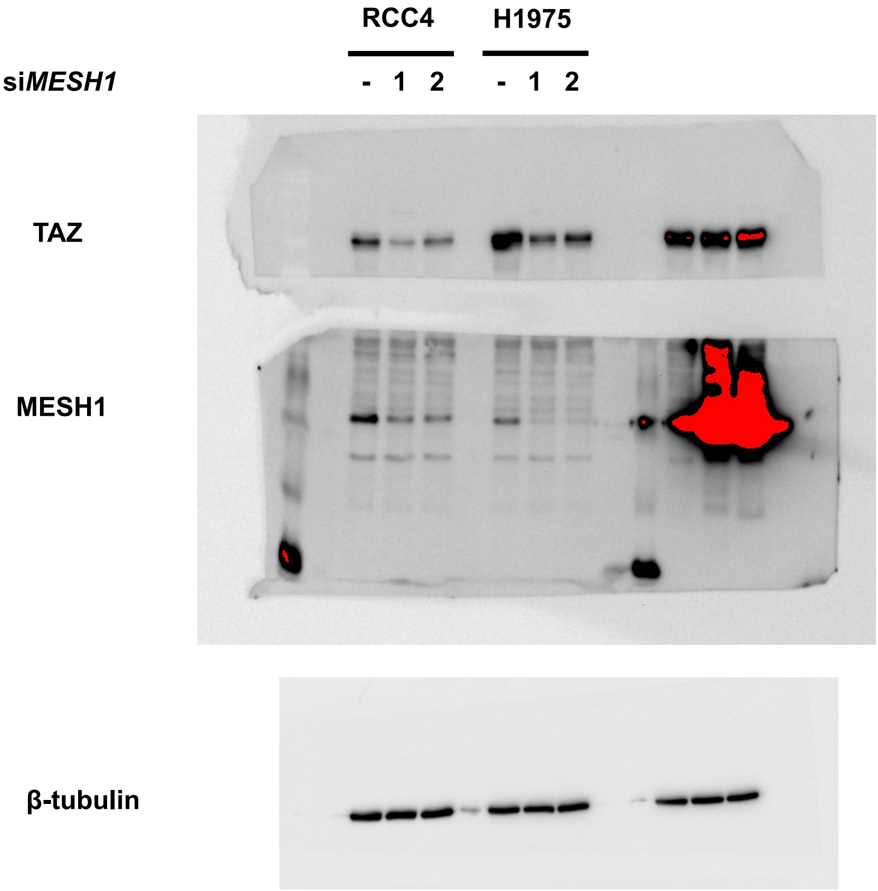

Figure 4

i

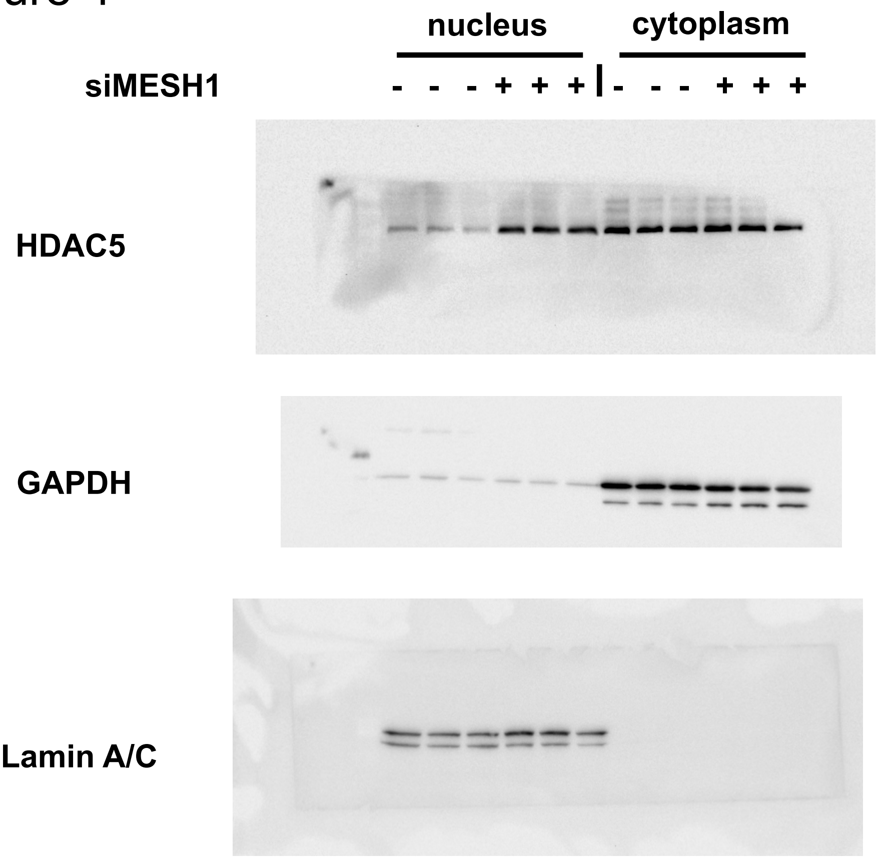

Figure 5

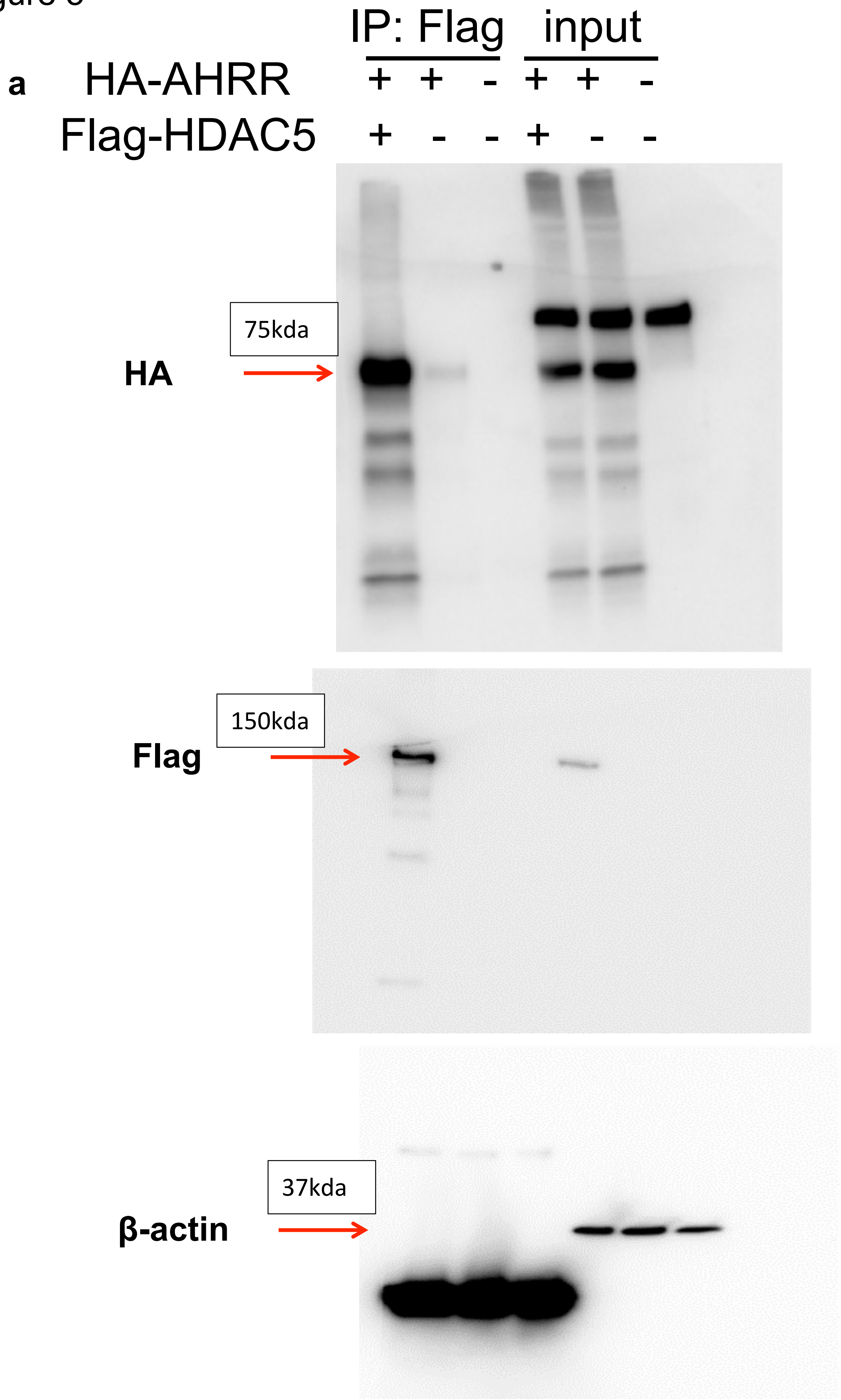

a
IP: Flag
input
HA-AHRR
+ + - + + -
Flag-HDAC5
+ - - + - -
HA
75kda
Flag
150kda
β-actin
37kda

Supplemental Fig 1

c

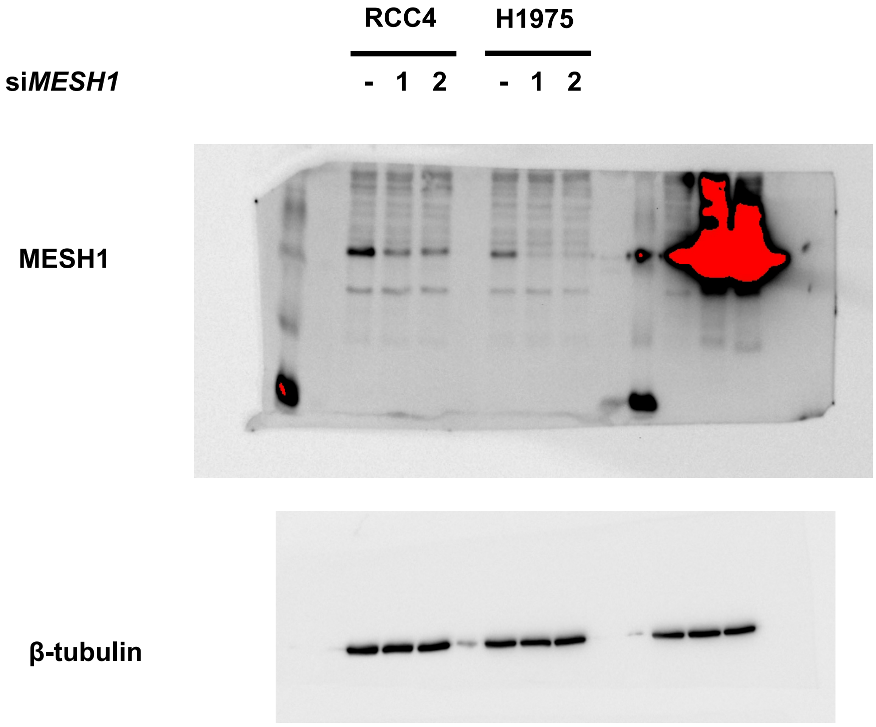

i

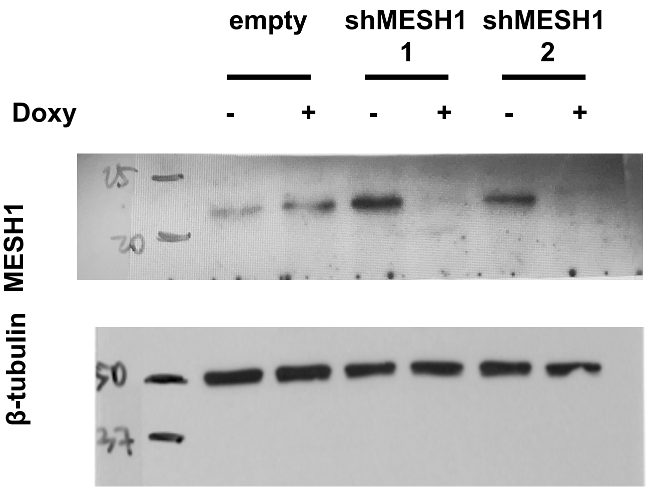

Supplemental Fig 3

e

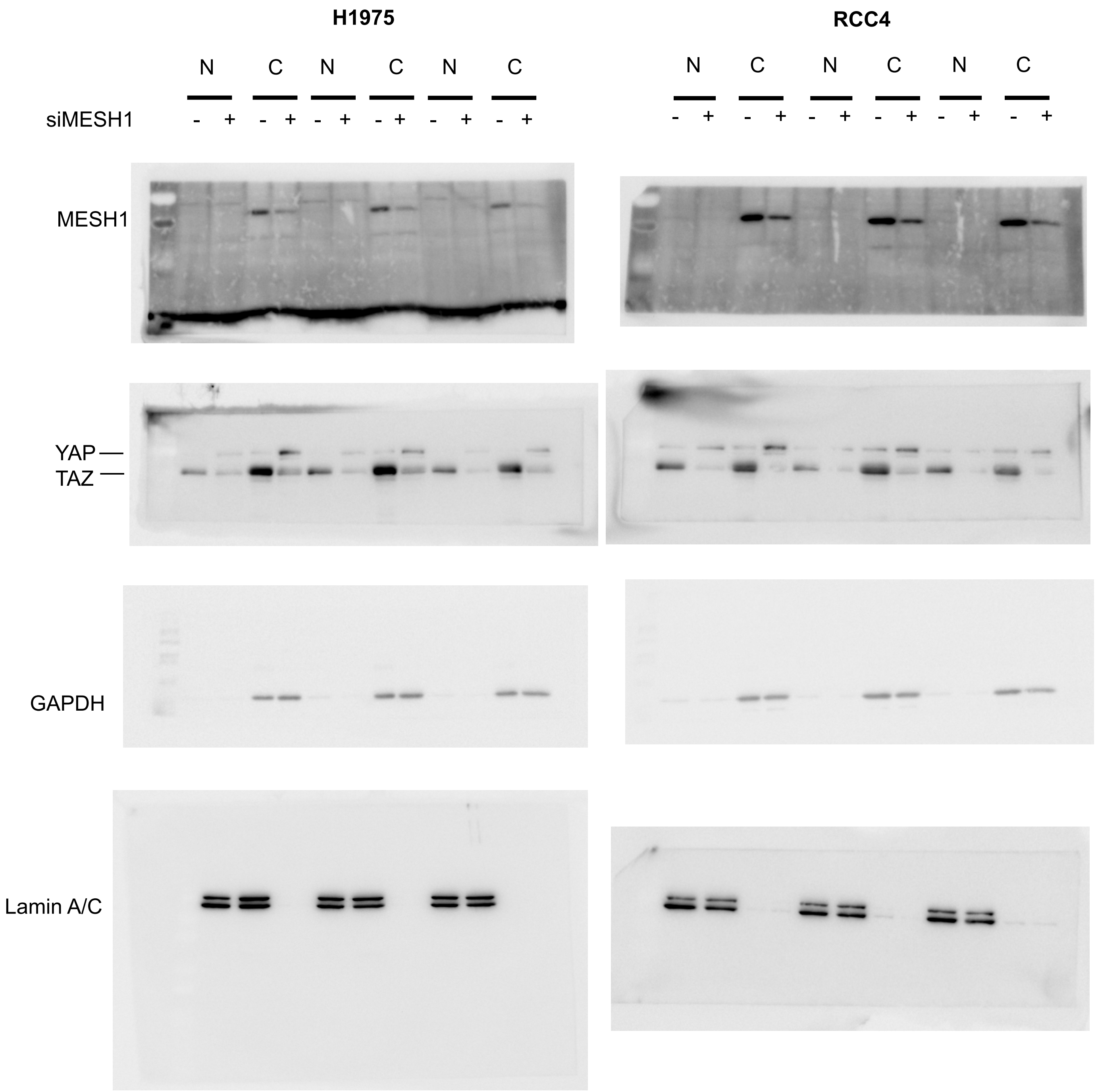

Supplemental Figure 4

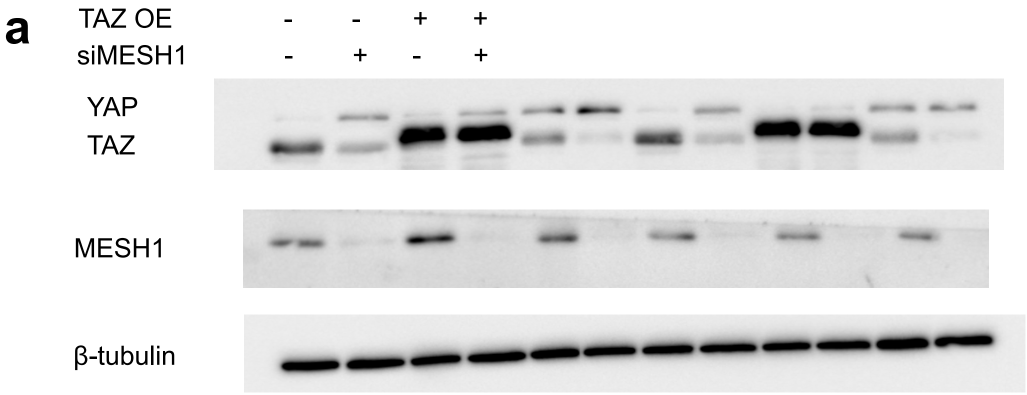

Supplemental Figure 5

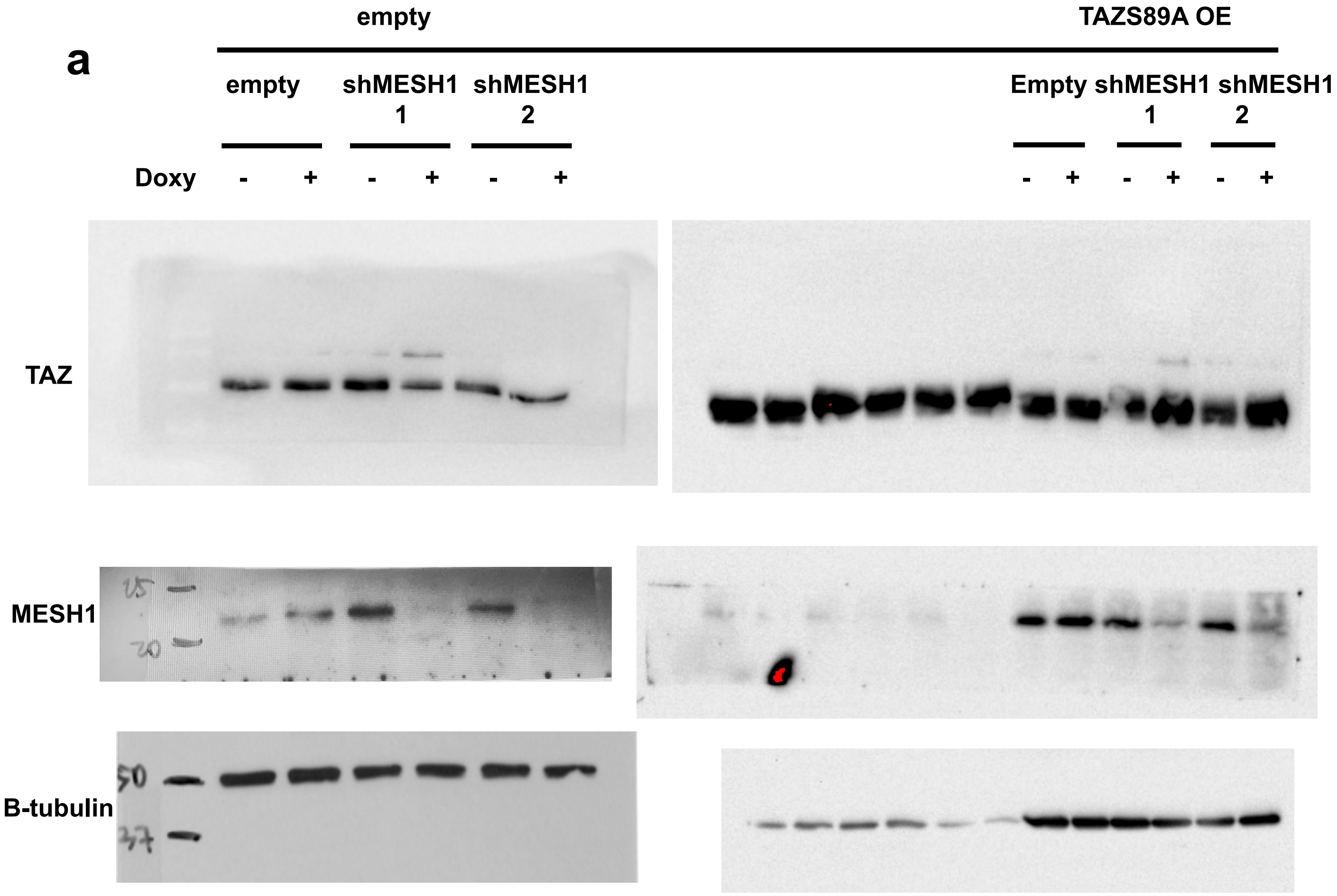

Supplemental Figure 7

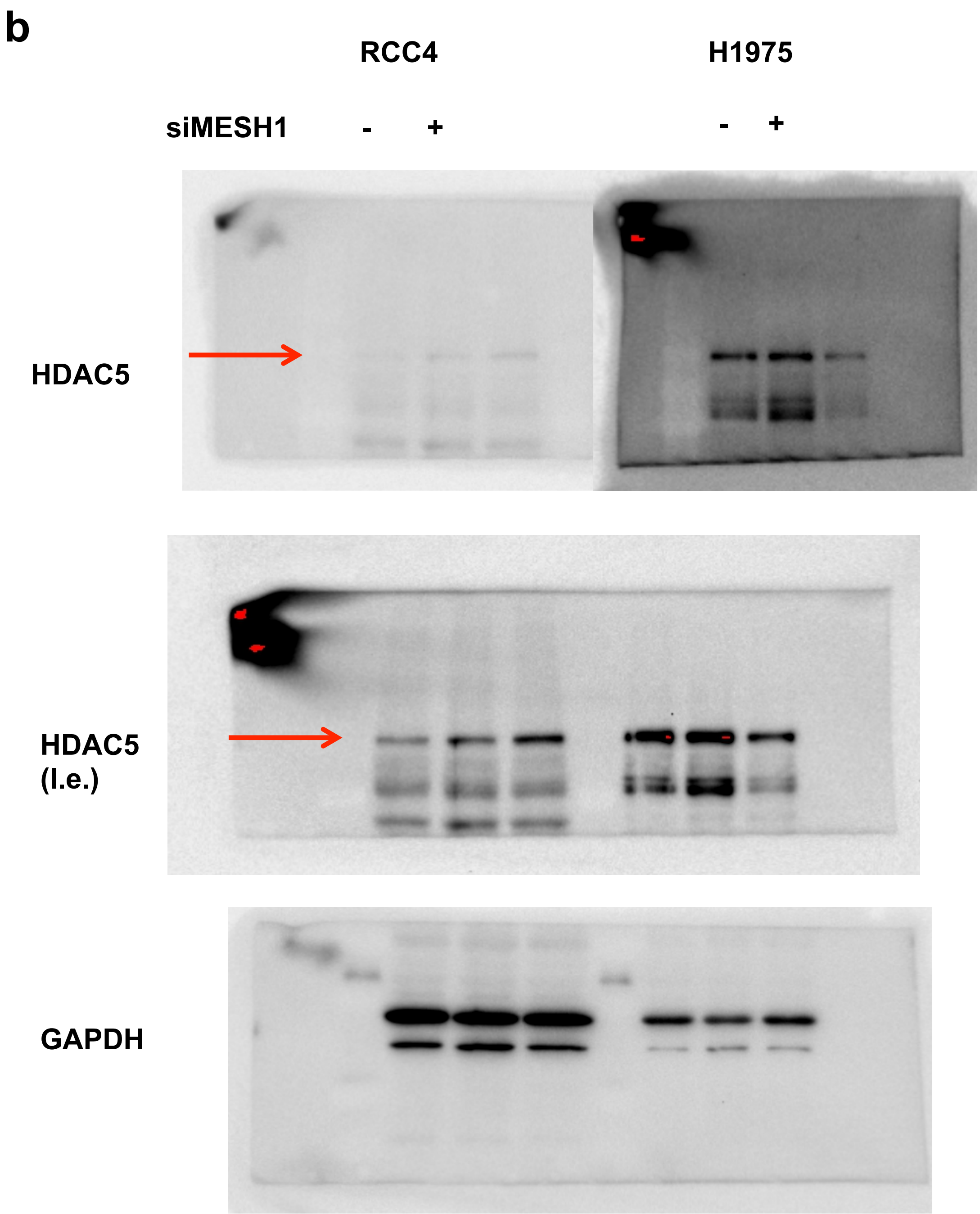

Supplement: Supplementary file 6 — Supplemental Material-original western blots [file 41419_2022_4663_MOESM6_ESM.pdf]
